# Direct Oxidation of Methane to Methanol over Transition-Metal-Free Ferrierite Zeolite Catalysts

**DOI:** 10.1021/jacs.4c00646

**Published:** 2024-04-01

**Authors:** Peipei Xiao, Yong Wang, Yao Lu, Kengo Nakamura, Nobuki Ozawa, Momoji Kubo, Hermann Gies, Toshiyuki Yokoi

**Affiliations:** † Nanospace Catalysis Unit, Institute of Innovative Research, Tokyo Institute of Technology, 4259 Nagatsuta, Midori-ku, Yokohama 226-8503, Japan; ‡ New Industry Creation Hatchery Center, Tohoku University, 6-6-10 Aoba, Aramaki, Aoba-ku, Sendai 980-8579, Japan; § Institute for Materials Research, Tohoku University, 2-1-1 Katahira, Aoba-ku, Sendai 980-8577, Japan; ∥ Institute of Geology, Mineralogy und Geophysics, Ruhr-University Bochum, Bochum 44780, Germany; ⊥ iPEACE223 Inc., Konwa Building, 1-12-22 Tsukiji, Chuo-ku, Tokyo 104-0045, Japan

## Abstract

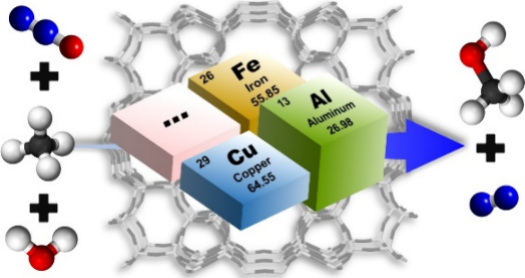

Direct oxidation of methane to methanol was reported to be highly dependent on the transition- or noble-metal-loading catalysts in the past decades. Here, we show that the transition-metal-free aluminosilicate ferrierite (FER) zeolite effectively catalyzed methane and N_2_O to methanol for the first time. The distorted tetracoordinated Al in the framework and pentacoordinated Al on the extra framework formed during calcination, activation, and reaction processes were confirmed as the potential active centers. The possible reaction pathway similar to the Fe-containing zeolites was advocated based on the reaction results using different oxidants, N_2_O adsorption FTIR spectra, and ^27^Al MAS NMR spectra. The stable and efficient methanol production capacity of FER zeolite was ascribed to the two-dimensional straight channels and its distinctive Al distribution of FER zeolite (CP914C) from Zeolyst. The transition-metal-free FER zeolite performed better than the record in the literature and our recent results using transition-metal-containing catalysts in terms of selectivity and formation rate of methanol and stability. This work has great significance and prospects for utilizing CH_4_ and N_2_O as resources and will open new avenues for methane oxidation.

## Introduction

Reduction and utilization of greenhouse gases, such as methane, nitrous oxide, and carbon dioxide, are the most important and urgent issues to be achieved.^1^ Catalytic conversion of these gas molecules into value-added chemicals has attracted a growing amount of attention. Among a variety of catalytic conversions of methane, direct conversion of methane into liquid chemicals suitable for transportation and storage, such as methanol, is a major subject with significant economic and environmental value.^2^ The current industrial route for methane to methanol is based on the indirect way that methane is first converted into syngas, a mixture of CO and H_2_, via an energy-intensive steam reforming process at high temperatures and then converted to other chemicals.^3^ To reduce time and economic cost, great efforts have been made in the direct routes including homogeneous solution phase,^4^ heterogeneous solution phase,^5^ and heterogeneous gas phase.^6^ However, many challenges, especially the ultralow methane conversion and methanol yield, still remain. Among the direct gas-phase route, the stepwise route has widely been used as a promising process because the high methanol selectivity was attained at relatively low temperatures (Table S1).^7^ However, the cumbersome operation process and low methanol yield are bottlenecks of this route. Correspondingly, the continuous route has the advantage of simple operation (Table S1).^8,9^ The subsequent tandem reactions made the diversity of products and reaction pathways from methane to hydrocarbons possible.^8,10^

Despite the reaction routes being diverse, the selection of catalysts was merely focused on precious metals such as Pt and Au^7,11^ and transition metals such as Cu, Co, and Fe as catalytically active species.^12^ Particularly, the transition-metal-exchanged zeolites have widely been used in both gas- and liquid-phase reaction systems.^12−14^ The single- or dual-nuclear transition-metal moieties have been reported as active sites,^15−17^ inspired by the natural biocatalytic enzyme systems.^18^ Furthermore, no transition-metal loading made the preparation of catalysts simpler and easier to obtain.

It is noteworthy, however, that the zeolite catalyst consisting of only main group elements for direct oxidation of methane to methanol (DMTM) has never been reported to date. FER is a 2-dimensional zeolite with 8-ring channels and intersected 10-ring channels.^19^ A cavity that is only accessible through the 8-ring windows is formed and known as the FER cage. FER zeolite is highly stable toward thermal, hydrothermal, and chemical treatments.^19^

Here, we reported the transition-metal-free aluminosilicate FER-type zeolite (CP914C, Zeolyst) (Figure S1 and Tables S2−S4) as a highly efficient catalyst to directly oxidize methane to methanol using N_2_O as the source of oxygen for the first time. The state-of-the-art 305 μmol g^–1^ min^–1^ methanol rate with 89% methanol and 10% DME selectivity was steadily achieved under the optimized reaction conditions (Figure S2), which was far beyond the record in the literature and our recent marks using transition-metal-containing zeolite catalysts (Table S1).^8,17,20−22^ The active sites were researched by combining the reaction performance of different FER zeolites with the ^27^Al MAS NMR spectra. The possible reaction pathway was mentioned based on the N_2_O adsorption FTIR spectra and reaction performance using different oxidants. Moreover, the reasons for the stable methanol production from methane were rationally explained from the perspective of the structure and Al distribution of the FER zeolite.

## Results and Discussion

### Effects of Reaction Conditions on the Reaction Performance

**Figure 1:**
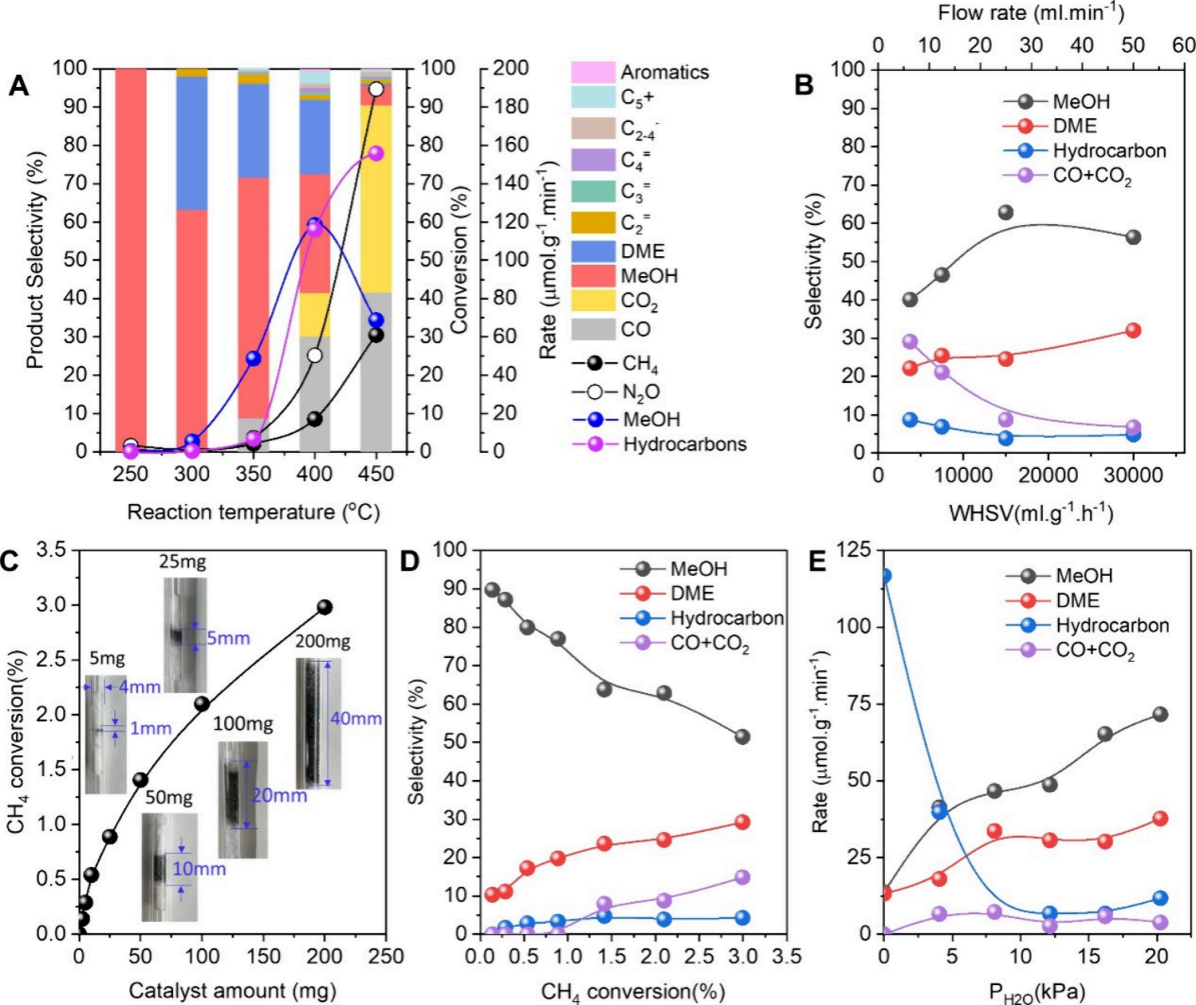
(A) Performance as a function of the reaction temperature at TOS = 0.17 h. Reaction conditions: 100 mg of H-FER catalyst, 250–450 °C, CH_4_/N_2_O/H_2_O/Ar = 10/10/2/3 mL min^–1^. (B) Product selectivity as a function of WHSV by changing the flow rate. Reaction conditions: 100 mg of H-FER catalyst, 350 °C. (C) CH_4_ conversion as a function of catalyst amount at TOS = 0.17 h. Reaction conditions: 2.5–200 mg of H-FER catalyst, 350 °C, CH_4_/N_2_O/H_2_O/Ar = 10/10/2/3 mL min^–1^. (D) Product selectivity as a function of CH_4_ conversion by adjustment of the catalyst amount. Reaction conditions: 2.5–200 mg of catalyst, 350 °C, CH_4_/N_2_O/H_2_O/Ar = 10/10/2/3 mL min^–1^. (E) Product formation rate as a function of water partial pressure at TOS = 0.17 h. Reaction conditions: 100 mg of H-FER catalyst, 350 °C. *r*_hydrocarbon_ = 2(*r*_C2_^=^ + *r*_C2_^–^) + 3(*r*_C3_^=^ + *r*_C3_^–^) + 4(*r*_C4_^=^ + *r*_C4_^–^) + 5(*r*_C5_^*=*^ + *r*_C5_^–^).

The proton form FER zeolite (H-FER) was activated in argon at 500 °C for 1 h; hereafter, both CH_4_ and N_2_O flow through the activated FER zeolite, followed by GC-TCD and GC-FID detectors of the mixture gas including the reactants and products (Figure S3). The effects of reaction conditions including temperature, total flow rate, catalyst amount, and H_2_O flow rate on the reaction performance were investigated (Figures S4–S11). With the reaction temperature increasing from 250 to 450 °C, the methanol selectivity was reduced from 100 to 5%, while the methanol formation rate was promoted from 0.4 μmol g^–1^ min^–1^ at 250 °C to 119 μmol g^–1^ min^–1^ at 400 °C and then dropped to 69 μmol g^–1^ min^–1^ at 450 °C (Figures 1A and S4–S8). The result illustrated that raising the reaction temperature was beneficial for providing more active centers. The selectivity of methanol and DME slightly increased along with the weight hourly space velocity (WHSV) from 3750 to 30000 mL g^–1^ h^–1^ by adjusting the total flow rate from 6.25 to 50 mL min^–1^. Correspondingly, the selectivity of hydrocarbons and CO_X_ (CO and CO_2_) declined with WHSV. The results indicated that increasing the total flow rate was able to decrease the mass transfer resistance (Figures 1B and S9).

Thereafter, the influence of the catalyst amount on the reaction performance was studied (Figures 1C and S10). Noteworthy, no catalyst meant no converted CH_4_. When the catalyst amount was varied from 2.5 to 25 mg, the methane conversion proportionally improved from 0.1 to 0.9%. However, when the catalyst amount was increased from 50 to 200 mg, the methane conversion moderately growth from 1.4 to 3.0%. The linear relationship between catalyst amount and methane conversion indicated that the reaction belonged to the kinetic control region, which was able to reflect the true catalytic activity. The nonlinear relationship meant that there was mass transfer resistance when the catalyst bed was longer than 5 mm, which was not entirely kinetic control (Figures 1C and S10). The relation of methane conversion with product selectivity was visible in Figure 1D, where the methane conversion was adjusted by changing the catalyst amount in Figure 1C. The methanol selectivity decreased from 90 to 51% with the methane conversion growing from 0.1 to 3.0% due to the secondary reactions on the backstage of the catalyst bed. The selectivity of products from the secondary reactions, including methanol to DME, methanol to hydrocarbons, and methanol to CO_*x*_, increased with methane conversion, which was consistent with the result in the literature.^9^ It is noteworthy that the secondary reaction of methanol to hydrocarbons was limited even with a high total acid amount of 1.01 mmol g^–1^ of FER zeolite (Table S2). One of the possible reasons was the two-dimensional (2D) and straight channels of FER zeolite, which avoided the mass transfer in channels.

Similar to the Cu-containing zeolite catalysts,^17,22,23^ the water participant notably influenced the methanol formation rate and the product distribution (Figures 1E and S11). With the water partial pressure rising from 0 to 20 kPa by changing the H_2_O flow rate from 0 to 5 mL min^–1^, the formation rate of methanol and DME was boosted from 14 to 72 μmol g^–1^ min^–1^ and from 14 to 38 μmol g^–1^ min^–1^, respectively. It should be emphasized that the total hydrocarbon formation rate was up to 117 μmol g^–1^ min^–1^ at the lowest water partial pressure (Figures 1E and S11). These facts suggest that methane was able to be directly oxidized to hydrocarbons via methanol/DME intermediates on the H-FER zeolite, while the participation of water limited the further conversion from methanol to hydrocarbons on the acid sites to a large extent. A similar phenomenon has been explained that H_2_O molecules could directly participate in methane oxidation through a proton-transfer route, which favors the formation of surface methoxy groups and accelerates methanol production.^17,22,23^ In addition, a higher water partial pressure was also conducive to dealumination, thus resulting in the increased amount of active Al sites and the reduced amount of acid sites. Analogous research has been implemented by Román-Leshkov and co-workers using Cu/CHA zeolite as catalyst and O_2_ as oxidant, where the lower water partial pressures resulted in more frequent interactions of activated C1 intermediates and thus the higher formation rate of CO_2_.^9^ However, in our work, the lower water partial pressure contributed to the higher formation rate of the hydrocarbon. The difference was ascribed to the diverse activity of O_2_ and N_2_O as oxidants.^24,25^

### Probing Active Sites

**Figure 2:**
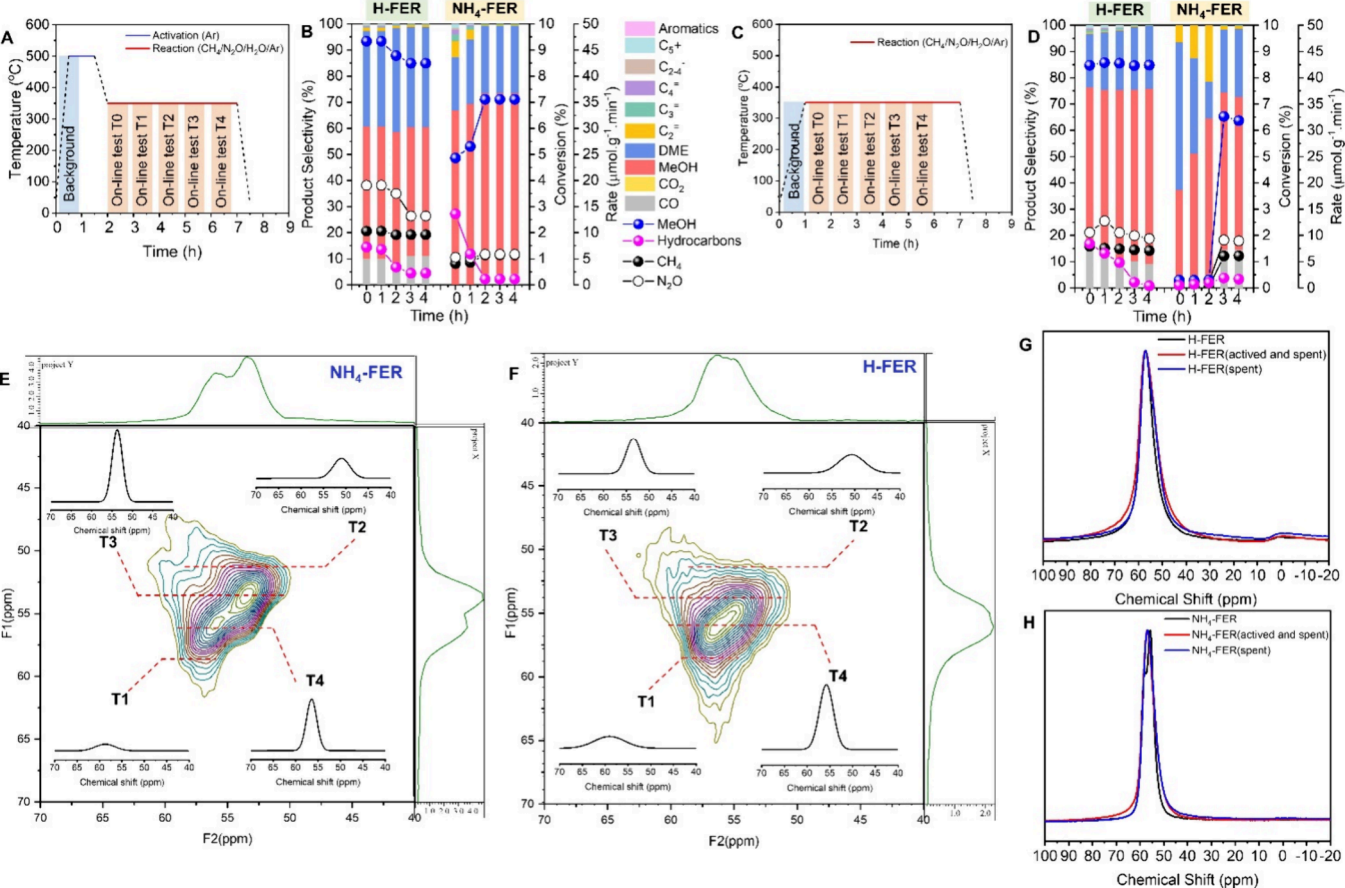
(A) Temperature program of activation at 500 °C for 1 h and then reaction at 350 °C. (B) Comparison of the reaction performance of H-FER and NH_4_-FER after activation at 500 °C for 1 h. Reaction conditions: 100 mg of catalyst, reaction temperature 350 °C, CH_4_/N_2_O/H_2_O/Ar = 10/10/2/3 mL min^–1^. (C) Temperature program of direct reaction without activation at 350 °C. (D) Comparison of reaction performance of H-FER and NH_4_-FER without activation. Reaction conditions: 100 mg of catalyst, reaction temperature 350 °C, CH_4_/N_2_O/H_2_O/Ar = 10/10/2/3 mL min^–1^. ^27^Al MQMAS NMR spectra of (E) NH_4_-FER and (F) H-FER zeolites with F1/F2 projections. Comparison of the ^27^Al MAS NMR spectra of fresh, after reaction with activation, and after reaction without activation for (G) H-FER and (H) NH_4_-FER.

To explore the active sites and the formation process, NH_4_-FER (the original CP914C from Zeolyst) and H-FER (CP914C calcined at 550 °C for 5 h) were tested under different activation conditions (Figure 2A,C). H-FER and NH_4_-FER exhibited a 47 μmol g^–1^ min^–1^ methanol rate with 51% selectivity and a 24 μmol g^–1^ min^–1^ methanol rate with 67% selectivity, respectively, after activation at 500 °C for 1 h (Figure 2A,B). Interestingly, without activation, the methanol rate of H-FER slightly decreased from 47 to 42 μmol g^–1^ min^–1^; however, that of NH_4_-FER severely dropped from 24 to 2 μmol g^–1^ min^–1^ with the selectivity of methanol declining from 67 to 37% (Figure 2D). To shed light on the reason, the Al population among the T-sites on the two samples was resolved by ^27^Al MQ MAS NMR via scanning different slices along the F1 dimension (Figure 2E,F). The T1–T4 sites showed isotropic chemical shifts (δiso) at around 59, 51, 54, and 56 ppm, respectively, by taking the centers of gravity in the F1 and F2 dimensions of the MQ MAS NMR spectra into account.^26^ It was obvious to see that the highest Al population of NH_4_-FER and H-FER was at the T3 and T4 site, respectively (Table S3). The different Al distribution was one of the possible reasons for the different reaction performances between NH_4_-FER and H-FER. Moreover, the ^27^Al MAS NMR spectra of the fresh and spent samples were compared (Figure 2G,H). The bands of tetracoordinated Al for H-FER were broadened compared to that of NH_4_-FER, indicating the existence of distorted tetracoordinated Al in H-FER at 40–50 and ∼63 ppm (Al_IV-1_ means the normal tetracoordinated Al; Al_IV-2_ means the distorted tetracoordinated Al) (Table S3).^27^ The distorted tetracoordinated Al as the shoulders of normal tetracoordinated Al for H-FER zeolite became wider after reaction with activation. However, it was less obvious for H-FER after the reaction without activation (Figure 2G). A similar phenomenon was observed on the NH_4_-FER zeolite (Figure 2H). The information indicated that the Al_IV-2_ species formed in the process of activation would work as active sites. Therefore, the methanol formation rate of NH_4_-FER with activation was higher than that without activation (Figure 2B,D). In addition, when the TOS was 3.7 h (Figure 2D), the methanol formation rate of NH_4_-FER without activation suddenly grew and stabilized at around 32 μmol g^–1^ min^–1^ due to the gradually formed active Al species during the reaction process.

Additionally, when H-FER zeolite was continuously reacted at elevated temperatures from 250 to 450 °C after activation at 500 °C for 1 h (Figure S12), methane conversion steadily rose from 0.01 to 33.1%. Similarly, when the H-FER zeolite was continuously reacted at 350 °C after activation at raising temperature from 400 to 800 °C for 1 h (Figure S13), the methane conversion and methanol formation rate were improved from 1.3 to 2.8% and from 35 to 66 μmol g^–1^ min^–1^, respectively (Figure S13B). These results also revealed that the active sites were formed during the activation and reaction process and that the higher temperatures were beneficial for the formation of active Al species owing to dealumination.

**Figure 3:**
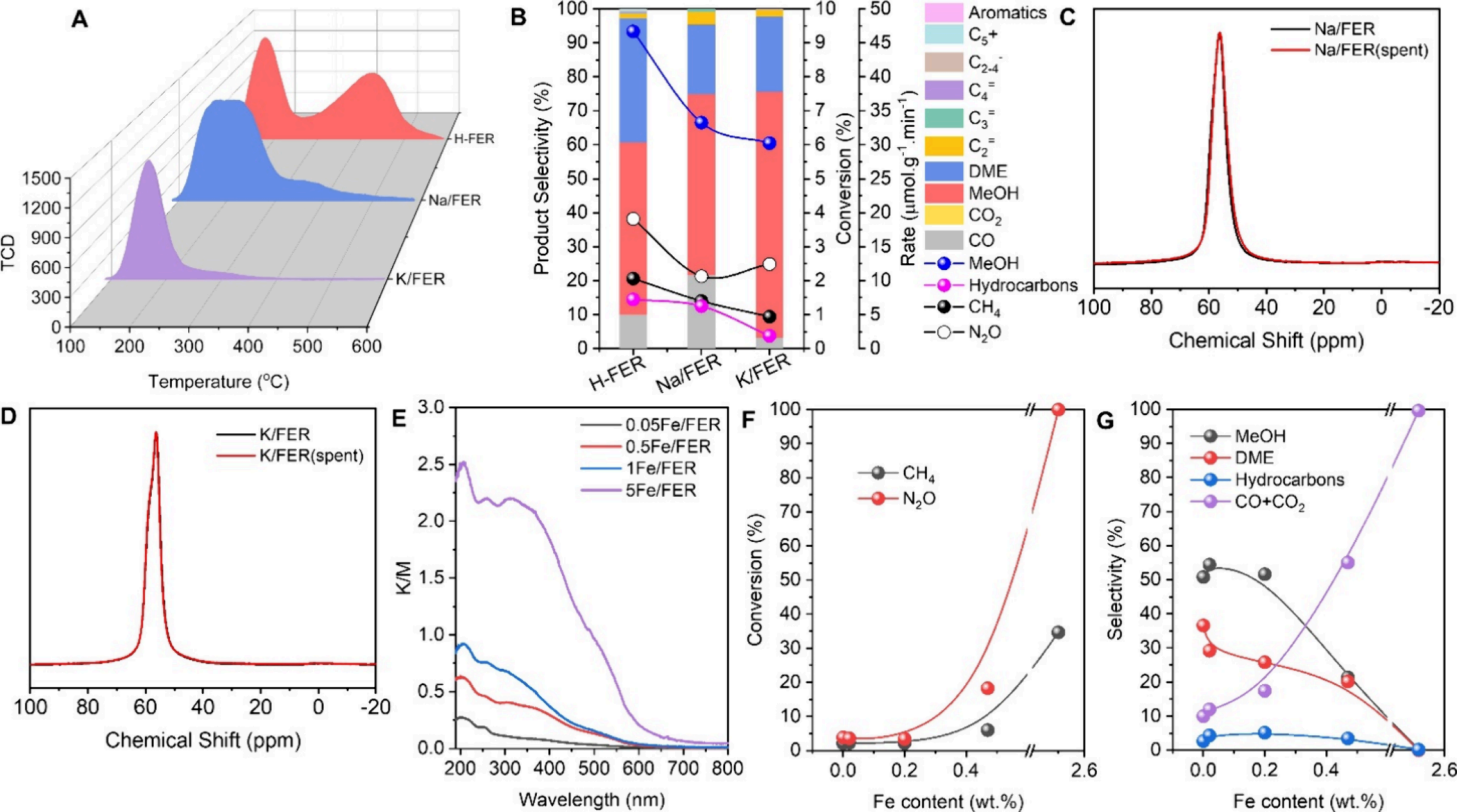
(A) Comparison of NH_3_-TPD patterns and (B) comparison of reaction performance of H-FER, Na/FER, and K/FER zeolite catalysts at TOS = 0.17 h. Comparison of the ^27^Al MAS NMR spectra of (C) fresh and spent Na/FER and (D) fresh and spent K/FER. (E) UV–vis spectra of *x*Fe/FER zeolites. (F) Conversion as a function of Fe content of *x*Fe/FER zeolites at TOS = 0.17 h. (G) Selectivity as a function of Fe content of *x*Fe/FER zeolites at TOS = 0.17 h. Reaction conditions: 100 mg of catalyst, 350 °C, CH_4_/N_2_O/H_2_O/Ar = 10/10/2/3 mL min^–1^.

Furthermore, to validate the relationship between the active site and the typical Bronsted acid sites (BAS) in zeolite, the H^+^ from Si(OH)Al was replaced by alkali metal cations Na^+^ or K^+^. The amount of BAS or strong acid sites of Na/FER (0.17 mmol g^–1^) and K/FER (0 mmol g^–1^) was remarkably decreased compared to H-FER (0.38 mmol g^–1^) (Figure 3A and Table S2). However, the methanol formation rates of Na/FER and K/FER were still up to 33 and 30 μmol g^–1^ min^–1^, respectively (Figures 3B and S14). This meant that BAS or a strong acid site was not the active center. Comparing the ^27^Al MAS NMR spectra of fresh and spent Na/FER and K/FER (Figure 3C,D), almost no variation was observed. It hinted that Na^+^ or K^+^ on the extra framework was able to stabilize Al in the framework, which has been reported by Hong and co-workers,^28,29^ thereby impeding the formation of active Al species.

Consider the high activity of Fe zeolites in the C–H bond activation with N_2_O.^30^ At first, we suspected that a trace amount of Fe was possibly contained in the commercial FER zeolite. However, based on the chemical composition of CP914C from Zeolyst and our measurement by ICP-AES, no Fe or Cu was detected. Moreover, the homemade aluminosilicate FER zeolite based on the literature^31^ was also active in direct oxidation of methane with N_2_O (Figures S15–S17). On the other hand, *x*Fe/FER zeolite catalysts with varying Fe content were prepared (Figures 3E and S18). Overall, the methane and N_2_O conversion increased with the Fe content growing (Figure 3F). However, when the Fe content was less than 0.02 wt %, the reactant conversion and methanol selectivity of Fe/FER were close to that of H-FER (Figure 3F,G). Only if the Fe content was high enough, here was higher than 0.47 wt %, the properties of Fe species were presented in the high N_2_O conversion and high CO_*x*_ selectivity (Figure 3F,G), which has been reported in our recent work.^13^ Additionally, the relation of the Cu content with selectivity was available (Figure S19). The high CO_X_ selectivity was offered even under the condition of very low Cu content (0.01 wt %). The selectivity of CO_X_ increased with Cu content due to the overoxidation of methanol to CO_*x*_ (Figure S19 and Table S2).^8,32,33^

**Figure 4:**
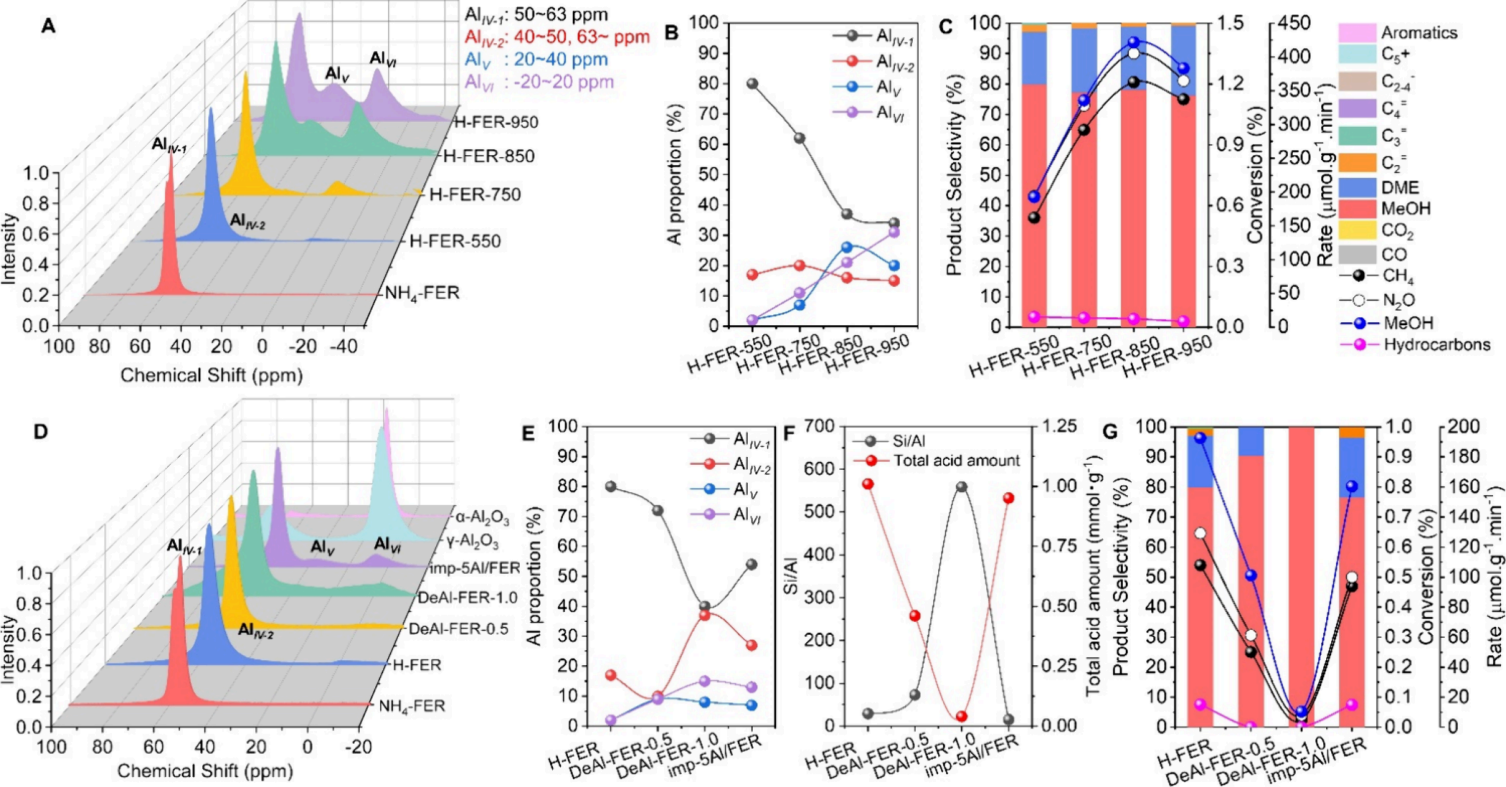
(A) ^27^Al MAS NMR spectra, (B) Al proportion, and (C) catalytic performance at TOS = 0.17 h of H-FER-*t* zeolites after calcination at different temperatures. Reaction conditions: 10 mg of catalyst, 350 °C, CH_4_/N_2_O/H_2_O/Ar = 10/10/2/3 mL min^–1^. (D) ^27^Al MAS NMR spectra, (E) Al proportion, (F) Si/Al ratio and total acid amount, and (G) catalytic performance of FER zeolites after dealumination and supplement Al by impregnation at TOS = 0.17 h. Reaction conditions: 10 mg of catalyst, 350 °C, CH_4_/N_2_O/H_2_O/Ar = 10/10/2/3 mL min^–1^.

To further confirm the active centers, the activities of different Al species were evaluated. The proportions of the normal tetracoordinated framework Al_IV-1_ (63–50 ppm), the distorted tetracoordinated framework Al_IV-2_ (50–40 and ∼63 ppm), the pentacoordinated extra framework Al_V_ (40–20 ppm), and the hexacoordinated extra framework Al_VI_ (20 to −20 ppm) were adjusted.^27,34^ First, the Al species was regulated by calcination at elevated temperature from 550 to 950 °C (Figures 4A and S20–S24, Tables S2–S4). The Al proportion of Al_IV-1_, Al_IV-2_, Al_V_, and Al_VI_ was visualized in Figure 4B. The changing trends of methane conversion and methanol formation rate (Figures 4C and S24) were consistent with that of Al_V_ (Figure 4B), possibly signifying that Al_V_ was a crucial component of the active centers.

To unearth the activity of other Al species, dealumination and replenishment of aluminum were implemented, respectively. Dealumination or precisely removing the aluminum at mild (denoted as “DeAl-FER-0.5”) and harsh (denoted as “DeAl-FER-1.0”) conditions was realized by using different concentrations of ammonium hexafluorosilicate (AHFS) solution (Figures 4D and S25–S29, Tables S2–S4).^35^ The severely surged Si/Al and declined acid amount confirmed the fact of dealumination (Figure 4E,F and Table S2). Concurrently, the severely diminished conversion and formation rate of DeAl-FER-0.5 and DeAl-FER-1.0 proved that the activity originated from Al (Figure 4F). Afterward, Al species was supplemented via impregnation method (denoted as “imp-5Al/FER”). The excess Al_2_O_3_ particles were not observed from the XRD pattern (Figure S30A). The Al_VI_ proportion of imp-5Al/FER was mainly improved and higher than that of H-FER (Figure 4E). However, the methane conversion and methanol formation rate of imp-5Al/FER were lower than those of H-FER (Figures 4G and S32). It revealed that Al_VI_ may not be the active center. It should be noted that the chemical shift of the supplied Al_VI_ was at 6.3 ppm^36^ and different from the Al_VI_ centered at 0 ppm formed by calcination (Figure 4A) but close to the chemical shift of γ-Al_2_O_3_ (10.3 ppm) and α-Al_2_O_3_ (14.7 ppm) (Figure 4D). Both γ-Al_2_O_3_ and α-Al_2_O_3_ did not show activity in the oxidation of methane with N_2_O (Figure S33). Conversely, the extra-added Al species on the extra framework might have an impact on the textural properties (Figure S30D) and the stability of framework Al, causing the reduced activity of imp-5Al/FER (Figure 4G).

### Reaction Mechanism

**Figure 5:**
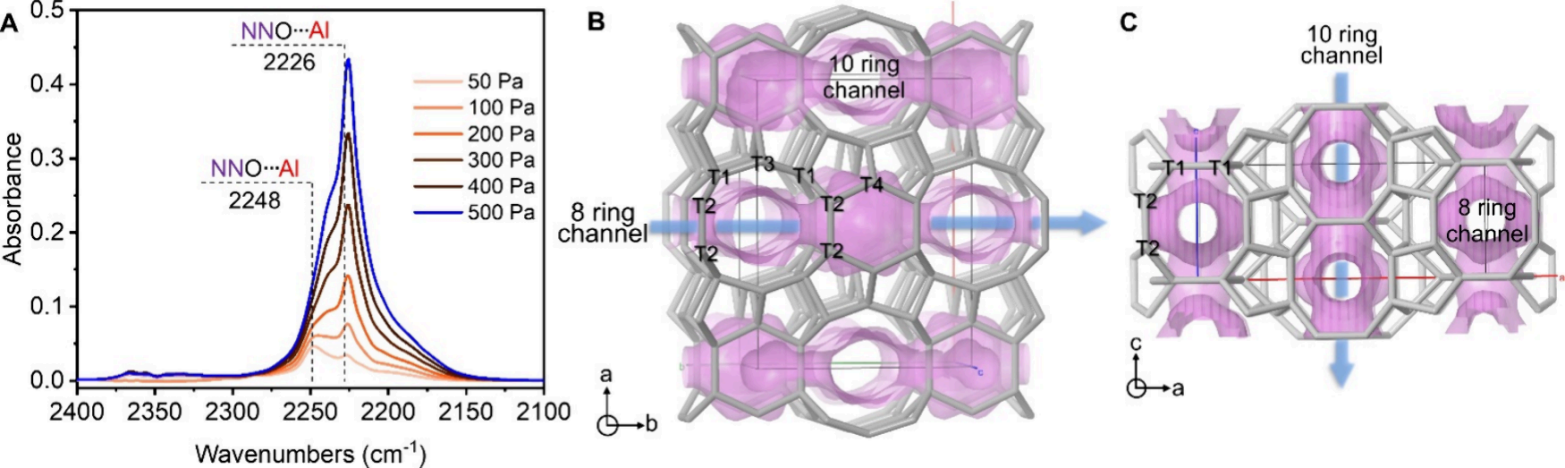
(A) N_2_O (50–500 Pa) adsorption FTIR spectra at 25 °C over H-FER zeolite catalyst after being evacuated at 500 °C for 1 h. Positions of four crystallographically independent T sites and the two perpendicular intersecting channels accessible via (B) 001 and (C) 010 faces.

Subsequently, the possible reaction route of direct oxidation of methane to methanol over an aluminosilicate zeolite was considered. The reaction performance of the H-FER zeolite using different oxidants, including N_2_O, O_2_, and H_2_O, was examined (Figure S34). Only the N_2_O-involved case obtained the products. These behaviors were quite consistent with the case of 5Fe/FER (Figure S35). However, 5Cu/FER was active using N_2_O or O_2_ as an oxidant (Figure S36). It signified that aluminosilicate zeolite and Fe-containing zeolite might have a similar reaction mechanism in oxidation of methane using N_2_O as the oxidant.^37,38^ Simultaneously, when N_2_O was adsorbed on H-FER zeolite, two bands belonging to Al-ONN at 2248 and 2226 cm^–1^ were observed in the FTIR spectrum (Figure 5A),^36^ which was similar to the Fe-containing zeolite but different from the Cu-containing zeolites that have been reported in our recent works.^13,20,39−41^ Thus, we concluded that the possible reaction pathway of aluminosilicate zeolite was similar to that of the Fe-containing zeolite. First N_2_O was adsorbed on the active Al sites as the Al-ONN motif. Subsequently, “α-O” formed after releasing N_2_, followed by the adsorption of CH_4_ on the active “α-O” sites and the breaking of C–H.^37,38,42^ Finally, CH_3_OH was desorbed, and active Al species were recovered. The apparent activation energy calculated at 270–300 °C based on the Arrhenius formula was 163 kJ mol^–1^ (Figure S37), which was close to the activation energy 142 ± 30 kJ mol^–1^ on Cu-SSZ-13 zeolite in the analogous conditions reported by Lobo and co-workers.^43^

### Explanation of Stability

Finally, the reasons for the efficient and stable production of methanol on the H-FER zeolite were elucidated. In the MTH reaction (Figure S38), the selectivity of C_5+_ was over 50%, indicating that the MTH reaction mainly occurred in the 10-ring channels and brought severe carbon deposition (7% weight loss after reaction 4 h) (Figure S39).^44^ Combined with the fact that the FER zeolite contained a 2D channel system intersected by 10- and 8-ring straight channels, once the intersection was blocked, the channels connected with the intersection were impassable (Figure S38B,C). This was the primary reason that FER zeolite was rapidly inactivated and rarely applied in MTH reaction. As regards the direct oxidation of methane to methanol reaction, methanol was possibly produced in most of the aluminosilicate zeolites. However, the activity and stability were greatly affected by zeolite structure and Al environment. Here, we just showed the reaction performance of ZSM-5 (JRC-Z5-30NH4) and MOR (JRC-Z-HM20) in the oxidation of methane at 300–450 °C (Figures S40 and S41, Table S2), which was much worse than that of FER zeolite. Additionally, the secondary reaction MTH was limited by the transfer path of methanol as well as the spatial distance between active Al sites and acid sites.^45−47^

The distribution of Al atoms in the framework, which directly affected the spatial distance between active Al sites and acid sites, is worth being pointed out. For the H-FER zeolite, ca. 64% Al belonged to T4 sites based on the deconvolution of ^27^Al MAS NMR spectrum (Figures 5B,C and S42, Table S3),^26,48^ which was only located in 8-ring channels. If methanol was formed on the active Al sites generated from T1 or T3, it was possible for it to be further converted at the acid sites of T2. However, the proportion of T2 was only 6%, resulting in the low possibility of methanol being converted to hydrocarbon at the acid sites of T2. Furthermore, the FER zeolite with altered Al distribution was synthesized based on the literature (named H-FER(Pyrr)) that has been mentioned above (Figures S15–S17 and Tables S2–S4).^31^ Specifically, the Al proportion of T4 decreased to 39% and T3 raised to 33% based on the deconvolution of the ^27^Al MAS NMR spectrum (Figure S16), and the initial hydrocarbon selectivity was up to 92% at 350 °C in the DMTM reaction (Figure S17). Among hydrocarbons, the selectivity of C_5_+ was up to 52%, indicating the secondary MTH reaction occurred in 10-ring channels. T1, T2, and T3 were located in the 10 MR intersection, and the Al proportion on these sites was up to ca. 52% among all the Al content (Figure S16B). In addition to affecting the secondary reaction, the Al distribution also affected the methanol formation rate. The methanol formation rate of H-FER(Pyrr) was around 17 mmol g^–1^ min^–1^ (Figure S17B), while under the same conditions, the value of H-FER was 47 mmol g^–1^ min^–1^. Briefly, the efficient and stable methanol production was attributed to the 2D channel system of the FER zeolite and the unique Al distribution of CP914C from Zeolyst, which was a perfect embodiment of the confinement reaction.

## Conclusions

In summary, the capability of direct oxidation of methane to methanol on the transition-metal-free aluminosilicate FER zeolite was reported for the first time. The bidirectional microporous structure of FER zeolite, in which 8- and 10-ring channels intersect at the FER cage, contributed to the efficient and stable oxidation of methane to methanol. Additionally, the particular Al distribution of the FER zeolite (CP914C, Zeolyst) successfully avoids a further cascade reaction of methanol to hydrocarbons. Distorted tetracoordinated Al in the framework and pentacoordinated Al on the extra framework were confirmed by ^27^Al MAS NMR spectra to be the potential active sites formed in the process of calcination, activation, and reaction process. A possible reaction pathway similar to the Fe/zeolite was claimed based on the reaction performance using different oxidants, N_2_O adsorption FTIR spectra, and ^27^Al MAS NMR spectra. These findings will open up a brand-new avenue for the development of direct oxidation of methane to methanol on transition-metal-free aluminosilicate zeolites, which enables the utilization of CH_4_ and N_2_O to other valuable chemicals on aluminosilicate zeolites with different topological structures.

## Supporting Information Available

Experimental details, methods, characterization results about XRD, ICP-AES, AAS, SEM images, N_2_ adsorption and desorption, NH_3_-TPD, TG-DTA, N_2_O and CH_4_ adsorption FTIR spectra, information on FER zeolite with different Al distribution, information on ZSM-5, MOR zeolites, reaction performance of other catalysts in direct oxidation of methane, reaction performance of FER zeolite in methanol to hydrocarbon reaction (PDF)

## References

[ref1] Schulze E. D.; Luyssaert S.; Ciais P.; Freibauer A.; Janssens I. A.; Soussana J. F.; Smith P.; Grace J.; Levin I.; Thiruchittampalam B.; Heimann M.; Dolman A. J.; Valentini R.; Bousquet P.; Peylin P.; Peters W.; Rödenbeck C.; Etiope G.; Vuichard N.; Wattenbach M.; Nabuurs G. J.; Poussi Z.; Nieschulze J.; Gash J. H. Importance of methane and nitrous oxide for Europe’s terrestrial greenhouse-gas balance. Nature Geoscience 2009, 2 (12), 842–850. 10.1038/ngeo686.

[ref2] Schwach P.; Pan X.; Bao X. Direct Conversion of Methane to Value-Added Chemicals over Heterogeneous Catalysts: Challenges and Prospects. Chem. Rev. 2017, 117 (13), 8497–8520. 10.1021/acs.chemrev.6b00715. 28475304

[ref3] Pan X.; Jiao F.; Miao D.; Bao X. Oxide–Zeolite-Based Composite Catalyst Concept That Enables Syngas Chemistry beyond Fischer–Tropsch Synthesis. Chem. Rev. 2021, 121, 6588–6609. 10.1021/acs.chemrev.0c01012. 34032417

[ref4] Gunsalus N. J.; Koppaka A.; Park S. H.; Bischof S. M.; Hashiguchi B. G.; Periana R. A. Homogeneous Functionalization of Methane. Chem. Rev. 2017, 117, 8521–8573. 10.1021/acs.chemrev.6b00739. 28459540

[ref5] Dummer N. F.; Willock D. J.; He Q.; Howard M. J.; Lewis R. J.; Qi G.; Taylor S. H.; Xu J.; Bethell D.; Kiely C. J.; Hutchings G. J. Methane Oxidation to Methanol. Chem. Rev. 2023, 123 (9), 6359–6411. 10.1021/acs.chemrev.2c00439. 36459432 PMC10176486

[ref6] Olivos-Suarez A. I.; Szécsényi À.; Hensen E. J. M.; Ruiz-Martinez J.; Pidko E. A.; Gascon J. Strategies for the Direct Catalytic Valorization of Methane Using Heterogeneous Catalysis: Challenges and Opportunities. ACS Catal. 2016, 6 (5), 2965–2981. 10.1021/acscatal.6b00428.

[ref7] Agarwal N.; Freakley S. J.; McVicker R. U.; Althahban S. M.; Dimitratos N.; He Q.; Morgan D. J.; Jenkins R. L.; Willock D. J.; Taylor S. H.; Kiely C. J.; Hutchings G. J. Aqueous Au-Pd colloids catalyze selective CH4 oxidation to CH3OH with O2 under mild conditions. Science 2017, 358 (6360), 223–227. 10.1126/science.aan6515. 28882995

[ref8] Xiao P.; Wang Y.; Lu Y.; De Baerdemaeker T.; Parvulescu A.-N.; Müller U.; De Vos D.; Meng X.; Xiao F.-S.; Zhang W.; Marler B.; Kolb U.; Gies H.; Yokoi T. Effects of Al distribution in the Cu-exchanged AEI zeolites on the reaction performance of continuous direct conversion of methane to methanol. Appl. Catal., B 2023, 325, 122395 10.1016/j.apcatb.2023.122395.

[ref9] Dinh K. T.; Sullivan M. M.; Narsimhan K.; Serna P.; Meyer R. J.; Dincă M.; Román-Leshkov Y. Continuous Partial Oxidation of Methane to Methanol Catalyzed by Diffusion-Paired Cu Dimers in Copper-Exchanged Zeolites. J. Am. Chem. Soc. 2019, 141, 11641–11650. 10.1021/jacs.9b04906. 31306002

[ref10] Dinh K. T.; Sullivan M. M.; Serna P.; Meyer R. J.; Román-Leshkov Y. Breaking the Selectivity-Conversion Limit of Partial Methane Oxidation with Tandem Heterogeneous Catalysts. ACS Catal. 2021, 11 (15), 9262–9270. 10.1021/acscatal.1c02187.

[ref11] Jin Z.; Wang L.; Zuidema E.; Mondal K.; Zhang M.; Zhang J.; Wang C.; Meng X.; Yang H.; Mesters C.; Xiao F.-S. Hydrophobic zeolite modification for in situ peroxide formation in methane oxidation to methanol. Science 2020, 367, 193–197. 10.1126/science.aaw1108. 31919221

[ref12] Ravi M.; Sushkevich V. L.; Knorpp A. J.; Newton M. A.; Palagin D.; Pinar A. B.; Ranocchiari M.; van Bokhoven J. A. Misconceptions and challenges in methane-to-methanol over transition-metal-exchanged zeolites. Nat. Catal. 2019, 2 (6), 485–494. 10.1038/s41929-019-0273-z.

[ref13] Xiao P.; Nakamura K.; Lu Y.; Huang J.; Wang L.; Osuga R.; Nishibori M.; Wang Y.; Gies H.; Yokoi T. One-Pot Synthesized Fe-AEI Zeolite Catalysts Contribute to Direct Oxidation of Methane. ACS Catal. 2023, 13, 16168–16178. 10.1021/acscatal.3c04675.

[ref14] Xiao P.; Wang Y.; Nishitoba T.; Kondo J. N.; Yokoi T. Selective oxidation of methane to methanol with H_2_O2 over an Fe-MFI zeolite catalyst using sulfolane solvent. Chem. Commun. (Camb) 2019, 55 (20), 2896–2899. 10.1039/C8CC10026H. 30702094

[ref15] Newton M. A.; Knorpp A. J.; Sushkevich V. L.; Palagin D.; van Bokhoven J. A. Active sites and mechanisms in the direct conversion of methane to methanol using Cu in zeolitic hosts: a critical examination. Chem. Soc. Rev. 2020, 49, 1449–1486. 10.1039/C7CS00709D. 32107517

[ref16] Knorpp A. J.; Pinar A. B.; Baerlocher C.; McCusker L. B.; Casati N.; Newton M. A.; Checchia S.; Meyet J.; Palagin D.; van Bokhoven J. A. Paired Copper Monomers in Zeolite Omega: The Active Site for Methane-to-Methanol Conversion. Angew. Chem. 2021, 133, 5918–5922. 10.1002/ange.202014030.

[ref17] Woertink J. S.; Smeets P. J.; Groothaert M. H.; Vance M. A.; Sels B. F.; Schoonheydt R. A.; Solomon E. I. A [Cu_2_O]2+ core in Cu-ZSM-5, the active site in the oxidation of methane to methanol. Proc. Natl. Acad. Sci. U. S. A. 2009, 106 (45), 18908–13. 10.1073/pnas.0910461106. 19864626 PMC2776445

[ref18] Wang V. C.; Maji S.; Chen P. P.; Lee H. K.; Yu S. S.; Chan S. I. Alkane Oxidation: Methane Monooxygenases, Related Enzymes, and Their Biomimetics. Chem. Rev. 2017, 117 (13), 8574–8621. 10.1021/acs.chemrev.6b00624. 28206744

[ref19] Margarit V. J.; Diaz-Rey M. R.; Navarro M. T.; Martinez C.; Corma A. Direct Synthesis of Nano-Ferrierite along the 10-Ring-Channel Direction Boosts Their Catalytic Behavior. Angew. Chem. 2018, 130, 3517–3521. 10.1002/ange.201711418. 29485242

[ref20] Xiao P.; Wang Y.; Nakamura K.; Lu Y.; De Baerdemaeker T.; Parvulescu A.-N.; Müller U.; De Vos D.; Meng X.; Xiao F.-S.; Zhang W.; Marler B.; Kolb U.; Osuga R.; Nishibori M.; Gies H.; Yokoi T. Highly Effective Cu/AEI Zeolite Catalysts Contribute to Continuous Conversion of Methane to Methanol. ACS Catal. 2023, 13, 11057–11068. 10.1021/acscatal.3c02271.

[ref21] Xiao P.; Wang Y.; Nakamura K.; Lu YA.; Kondo J. N.; Gies H.; Yokoi T. c-Axis-oriented sheet-like Cu/AEI zeolite contributes to continuous direct oxidation of methane to methanol. Catal. Sci. Technol. 2023, 13, 5831–5841. 10.1039/D3CY00584D.

[ref22] Xu R.; Liu N.; Dai C.; Li Y.; Zhang J.; Wu B.; Yu G.; Chen B. H_2_O-Built Proton Transfer Bridge Enhances ContinuousMethane Oxidation to MethanoloverCu-BEA Zeolite. Angew. Chem. 2021, 60, 16634–16640. 10.1002/anie.202105167. 33982395

[ref23] Liu Z.; Huang E.; Orozco I.; Liao W.; Palomino R. M.; Rui N.; Duchon T.; Nemsak S.; Grinter D. C.; Mahapatra M.; Liu P.; Rodriguez J. A.; Senanayake S. D. Water-promoted interfacial pathways in methane oxidation to methanol on a CeO_2_-Cu_2_O catalyst. Science 2020, 368, 513–517. 10.1126/science.aba5005. 32355028

[ref24] Aydin Z.; Zanina A.; Kondratenko V. A.; Rabeah J.; Li J.; Chen J.; Li Y.; Jiang G.; Lund H.; Bartling S.; Linke D.; Kondratenko E. V. Effects of N2O and Water on Activity and Selectivity in the Oxidative Coupling of Methane over Mn–Na2WO4/SiO_2_: Role of Oxygen Species. ACS Catal. 2022, 12 (2), 1298–1309. 10.1021/acscatal.1c04915.

[ref25] Dasireddy V. D. B. C.; Hanzel D.; Bharuth-Ram K.; Likozar B. The effect of oxidant species on direct, non-syngas conversion of methane to methanol over an FePO4 catalyst material. RSC Adv. 2019, 9, 30989 10.1039/C9RA02327E. 35529365 PMC9072299

[ref26] Xiong Z.; Qi G.; Bai L.; Zhan E.; Chu Y.; Xu J.; Ta N.; Hao A.; Deng F.; Shen W. Preferential population of Al atoms at the T4 site of ZSM-35 for the carbonylation of dimethyl ether. Catal. Sci. Technol. 2022, 12 (16), 4993–4997. 10.1039/D2CY01112C.

[ref27] Chen K.; Gan Z.; Horstmeier S.; White J. L. Distribution of Aluminum Species in Zeolite Catalysts: 27Al NMR of Framework, Partially-Coordinated Framework, and Non-Framework Moieties. J. Am. Chem. Soc. 2021, 143, 6669–6680. 10.1021/jacs.1c02361. 33881305 PMC8212420

[ref28] Auepattana-Aumrung C.; Marquez V.; Wannakao S.; Jongsomjit B.; Panpranot J.; Praserthdam P. Role of Al in Na-ZSM-5 zeolite structure on catalyst stability in butene cracking reaction. Sci. Rep. 2020, 10 (1), 13643 10.1038/s41598-020-70568-z. 32788643 PMC7424521

[ref29] Ryu T.; Ahn N. H.; Seo S.; Cho J.; Kim H.; Jo D.; Park G. T.; Kim P. S.; Kim C. H.; Bruce E. L.; Wright P. A.; Nam I. S.; Hong S. B. Fully Copper-Exchanged High-Silica LTA Zeolites as Unrivaled Hydrothermally Stable NH(3) -SCR Catalysts. Angew. Chem., Int. Ed. Engl. 2017, 56 (12), 3256–3260. 10.1002/anie.201610547. 28097753

[ref30] Pirutko L. V.; Chernyavsky V. S.; Uriarte A. K.; Panov G. I. Oxidation of benzene to phenol by nitrous oxide Activity of iron in zeolite matrices of various composition. Appl. Catal. A-Gen 2002, 227, 143–157. 10.1016/S0926-860X(01)00932-2.

[ref31] Dai W.; Ruaux V.; Deng X.; Tai W.; Wu G.; Guan N.; Li L.; Valtchev V. Synthesis and catalytic application of nanorod-like FER-type zeolites. J. Mater. Chem. A 2021, 9 (44), 24922–24931. 10.1039/D1TA06596C.

[ref32] Zhao G.; Chodyko C.; Benhelal E.; Adesina A.; Kennedy E.; Stockenhuber M. Methane oxidation by N2O over Fe-FER catalysts prepared by different methods: Nature of active iron species, stability of surface oxygen species and selectivity to products. J. Catal. 2021, 400, 10–19. 10.1016/j.jcat.2021.04.019.

[ref33] Tao L.; Lee I.; Khare R.; Jentys A.; Fulton J. L.; Sanchez-Sanchez M.; Lercher J. A. Speciation of Cu-Oxo Clusters in Ferrierite for Selective Oxidation of Methane to Methanol. Chem. Mater. 2022, 34 (10), 4355–4363. 10.1021/acs.chemmater.1c04249.

[ref34] Ravi M.; Sushkevich V. L.; van Bokhoven J. A. Towards a better understanding of Lewis acidic aluminium in zeolites. Nat. Mater. 2020, 19 (10), 1047–1056. 10.1038/s41563-020-0751-3. 32958864

[ref35] Li C.; Guo L.; Liu P.; Gong K.; Jin W.; Li L.; Zhu X.; Liu X.; Shen B. Defects in AHFS-dealuminated Y zeolite: A crucial factor for mesopores formation in the following base treatment procedure. Microporous Mesoporous Mater. 2018, 255, 242–252. 10.1016/j.micromeso.2017.07.046.

[ref36] van der Bij H. E.; Cicmil D.; Wang J.; Meirer F.; de Groot F. M.; Weckhuysen B. M. Aluminum-phosphate binder formation in zeolites as probed with X-ray absorption microscopy. J. Am. Chem. Soc. 2014, 136 (51), 17774–87. 10.1021/ja508545m. 25415849

[ref37] Bols M. L.; Snyder B. E. R.; Rhoda H. M.; Cnudde P.; Fayad G.; Schoonheydt R. A.; Van Speybroeck V.; Solomon E. I.; Sels B. F. Coordination and activation of nitrous oxide by iron zeolites. Nat. Catal. 2021, 4 (4), 332–340. 10.1038/s41929-021-00602-4.

[ref38] Snyder B. E. R.; Bols M. L.; Rhoda H. M.; Plessers D.; Schoonheydt R. A.; Sels B. F.; Solomon E. I. Cage effects control the mechanism of methane hydroxylation in zeolites. Science 2021, 373, 327–331. 10.1126/science.abd5803. 34437151 PMC10353845

[ref39] Fanson P. T.; Stradt M. W.; Lauterbach J.; Delgass W. N. The effect of Si/Al ratio and copper exchange level on isothermal kinetic rate oscillations for N2O decomposition over Cu-ZSM-5: a transient FTIR study. Appl. Catal., B 2002, 38, 331–347. 10.1016/S0926-3373(02)00073-5.

[ref40] Rac V.; Rakić V.; Damjanović-Vasilić L.; Dondur V.; Auroux A. Complementary approach to the adsorption of CO and N2O on bimetallic ion exchanged ZMS-5 zeolite: Microcalorimetric and FTIR spectroscopy study. Appl. Surf. Sci. 2017, 423, 1134–1140. 10.1016/j.apsusc.2017.06.269.

[ref41] Nobukawa T.; Yoshida M.; Kameoka S.; Ito S.-i.; Tomishige K.; Kunimori K. In-Situ Observation of Reaction Intermediate in the Selective Catalytic Reduction of N2O with CH4over Fe Ion-Exchanged BEA Zeolite Catalyst for the Elucidation of Its Reaction Mechanism Using FTIR. J. Phys. Chem. B 2004, 108 (13), 4071–4079. 10.1021/jp030867u.

[ref42] Mahyuddin M. H.; Staykov A.; Shiota Y.; Miyanishi M.; Yoshizawa K. Roles of Zeolite Confinement and Cu–O–Cu Angle on the Direct Conversion of Methane to Methanol by [Cu2(μ-O)]2+-Exchanged AEI, CHA, AFX, and MFI Zeolites. ACS Catal. 2017, 7 (6), 3741–3751. 10.1021/acscatal.7b00588.

[ref43] Ipek B.; Lobo R. F. Catalytic conversion of methane to methanol on Cu-SSZ-13 using N2O as oxidant. Chem. Commun. (Camb) 2016, 52 (91), 13401–13404. 10.1039/C6CC07893A. 27790665

[ref44] Zhang M.; Xu S.; Wei Y.; Li J.; Chen J.; Wang J.; Zhang W.; Gao S.; Li X.; Wang C.; Liu Z. Methanol conversion on ZSM-22, ZSM-35 and ZSM-5 zeolites: effects of 10-membered ring zeolite structures on methylcyclopentenyl cations and dual cycle mechanism. RSC Adv. 2016, 6 (98), 95855–95864. 10.1039/C6RA08884H.

[ref45] Li Y.; Wang M.; Liu S.; Wu F.; Zhang Q.; Zhang S.; Cheng K.; Wang Y. Distance for Communication between Metal and Acid Sites for Syngas Conversion. ACS Catal. 2022, 12 (15), 8793–8801. 10.1021/acscatal.2c02125.

[ref46] Zečević J.; Vanbutsele G.; de Jong K. P.; Martens J. A. Nanoscale intimacy in bifunctional catalysts for selective conversion of hydrocarbons. Nature 2015, 528, 245–248. 10.1038/nature16173. 26659185 PMC4928701

[ref47] Cheng K.; Smulders L. C. J.; van der Wal L. I.; Oenema J.; Meeldijk J. D.; Visser N. L.; Sunley G.; Roberts T.; Xu Z.; Doskocil E.; Yoshida H.; Zheng Y.; Zečević J.; de Jongh P. E.; de Jong K. P. Maximizing noble metal utilization in solid catalysts by control of nanoparticle location. Science 2022, 377, 204–208. 10.1126/science.abn8289. 35857537

[ref48] Pinar A. B.; Rzepka P.; Knorpp A. J.; McCusker L. B.; Baerlocher C.; Huthwelker T.; van Bokhoven J. A. Pinpointing and Quantifying the Aluminum Distribution in Zeolite Catalysts Using Anomalous Scattering at the Al Absorption Edge. J. Am. Chem. Soc. 2021, 143 (43), 17926–17930. 10.1021/jacs.1c06925. 34695360

