# Supplementary material for: Direct Oxidation of Methane to Methanol over Transition-Metal-Free Ferrierite Zeolite Catalysts
Source: J Am Chem Soc. 2024 Apr 1;146(14):10014–22. doi: 10.1021/jacs.4c00646 (PMC11009945; doi:10.1021/jacs.4c00646)
Supplement: Supplementary file 1 — ja4c00646_si_001.pdf [file ja4c00646_si_001.pdf]

Supplementary Information

Direct oxidation of methane to methanol over transition-metal-free Ferrierite zeolite catalysts

Peipei Xiao,[a] Yong Wang,[a] Yao Lu,[a] Kengo Nakamura,[a] Nobuki Ozawa,[b,c] Momoji Kubo,[b,c]

Hermann Gies,[a,d] Toshiyuki Yokoi*[a,e]

[a] Nanospace Catalysis Unit, Institute of Innovative Research, Tokyo Institute of Technology, 4259 Nagatsuta, Midori-ku, Yokohama 226-8503, Japan

[b] New Industry Creation Hatchery Center, Tohoku University, 6-6-10 Aoba, Aramaki, Aoba-ku, Sendai 980-8579, Japan.

[c] Institute for Materials Research, Tohoku University, 2-1-1 Katahira, Aoba-ku, Sendai 980-8577, Japan.

[d] Institute of Geology, Mineralogy und Geophysics, Ruhr-University Bochum, Bochum 44780, Germany

[e] iPEACE223 Inc., Konwa Building, 1-12-22 Tsukiji, Chuo-ku, Tokyo, 104-0045, Japan

*E-mail: yokoi@cat.res.titech.ac.jp

Supplementary Methods

Catalysts Preparation

H-FER-*t* zeolite catalysts were prepared by calcination NH_4-FER (CP914C, Zeolyst) zeolite at 550, 750, 850, and 950 °C in air for 5 h, respectively, where *t* meant the calcination temperature. H-FER and H-FER-550 were the same sample.

Na/FER and K/FER zeolite catalysts were prepared by exchanging the NH_4-FER (CP914C, Zeolyst) zeolite with 2500 mmol/L $NaNO_3$ solution and 500 mmol/L KNO_3 solution, respectively, at a 100 mL/g liquid-to-solid ratio stirring at 80 °C for 24 h. The suspensions were filtered, washed, dried at 100 °C overnight, and calcined at 550 °C in air for 5 h.

*x*Fe/FER zeolite catalysts were prepared by exchanging the NH_4-FER (CP914C, Zeolyst) zeolite with 0.05, 0.5, 1, 5 mmol/L $Fe(NO_3)_3$ solution at a 100 mL/g liquid-to-solid ratio stirring at 80 °C for 24 h. The suspensions were filtered, washed, dried at 100 °C overnight, and calcined at 550 °C in air for 5 h. *x* meant the concentration of $Fe(NO_3)_3$ solution.

*y*Cu/FER zeolite catalysts were prepared by exchanging the NH_4-FER (CP914C, Zeolyst) zeolite with 0.1, 1, 5, 50 mmol/L $Cu(NO_3)_2$ solution at a 100 mL/g liquid-to-solid ratio stirring at 80 °C for 24 h. The suspensions were filtered, washed, dried at 100 °C overnight, and calcined at 550 °C in air for 5 h. *y* meant the concentration of $Cu(NO_3)_2$ solution.

Imp-5Al/FER zeolite catalyst was prepared by mixing 1 g NH_4-FER (CP914C, Zeolyst) zeolite with 5 ml aqueous solution containing 0.19 g $Al(NO_3)_3 \cdot 9H_2O$, and evaporating water at 80 °C, then drying overnight at 100 °C. Finally, the sample was calcined at 550 °C for 5 h. The calcined sample was remarked as imp-5Al/FER, where 5 meant the moles of added Al (0.5 mmol) in the impregnation process.

DeAl-FER zeolite catalysts were prepared by dealumination the NH_4-FER (CP914C, Zeolyst) zeolite using 0.5 and 1.0 mol/L Ammonium Hexafluorosilicate (AHFS) solution at a 100 mL/g liquid-to-solid ratio stirring at 80 °C for 24 and 48 h, respectively. The suspension was filtered, washed, and dried at 100 °C overnight. Afterward, the sample was exchanged twice with 2.5 M NH_4NO_3 aqueous solution at 80 °C for 3 h to get the NH_4-form samples. The suspension was filtered, washed, dried at 100 °C overnight, and calcined at 550 °C in air for 5 h. The final obtained samples were named as DeAl-FER-0.5 and DeAl-FER-1.0, respectively, where 0.5 and 1.0 meant the concentration of AHFS.

The homemade FER sample was synthesized refer to literature.[1] Specifically, a clearly basic solution with the composition of SiO_2: $0.05Al_2O_3$: $0.08Na_2O$: 0.6Pyrr: $20H_2O$ was first obtained by dissolving pyrrolidine, NaOH, and $NaAlO_2$ in deionized water, and then colloidal silica was added to the liquid and vigorously stirred at room temperature for 1 h. Finally, the obtained gel was transferred into an autoclave and subjected to crystallization in a tumbling oven (20 rpm) at 180 °C for 4 days. The solid product was collected by centrifugation, washed with deionized water until a near-neutral pH, and dried overnight at 100 °C. The as-synthesized sample was calcined at 550 °C for 10 h to remove the OSDA and then exchanged with 2.5 $mol \cdot L^{-1}$ NH_4NO_3 at 80 °C for 3 h. The NH_4-type FER sample was filtered, washed, and dried at 100 °C overnight. Finally, the NH_4-type FER sample was calcined in air at 550 °C for 5 h. The finally obtained sample was denoted as H-FER(Pyrr).

H-ZSM-5 zeolite catalyst was prepared by calcination the ZSM-5 (JRC-Z5-30NH4) zeolite in air at 550 °C for 5 h.

H-MOR zeolite catalyst was prepared by calcination the MOR (JRC-Z-HM20) zeolite in air at 550 °C for 5 h.

Catalysts characterization

^{27}Al MAS NMR

Solid-state ^{27}Al MAS NMR spectra were measured on a JEOL ECA-600 spectrometer at a resonance frequency of 156.4 MHz using a 4 mm sample rotor with a spinning rate of 15.0 kHz. The ^{27}Al chemical shift was referenced to -0.54 ppm of $AlNH_4(SO_4)_2 \cdot 12H_2O$.

^{27}Al MQMAS NMR

^{27}Al MQMAS NMR spectra were measured on the same equipment and obtained using three pulse sequence employing a z-filter. The triple quantum excitation and conversion pulse lengths were optimized to 3.4 and 1.8 μs, respectively, and the length of the selective pulse was 15.0 μs. A series of 512 t1 slices were collected using 2000 FIDs with a recycle delay of 0.1 s for each slice. The ^{27}Al single pulse MAS spectra were decomposed into the three components and the fitting values, the averaged isotropic chemical shift δ_{iso} the width of the Gaussian distribution of δ_{iso} (ΔCS), and the averaged quadrupolar coupling constant CQ were determined using the ‘‘Dmfit’’ program applying a simple Czjzek model.

NH_3-TPD

Temperature-programmed ammonia desorption (NH_3-TPD) profiles were recorded on Multitrack TPD equipment (Japan BEL). Typically, 25 mg of catalyst was pretreated at 600 °C in He (50 mL min^{-1}) for 1 h and then cooled to 100 °C. Prior to the adsorption of NH_3, the sample was evacuated at 100 °C for 1 h. Approximately 2500 Pa of NH_3 was allowed to contact with the sample at 100 °C for 10 min. Subsequently, the sample was evacuated to remove weakly adsorbed NH_3 at the same temperature for 30 min. Finally, the sample was cooled to 100 °C and heated from 100 to 600 °C at a ramping rate of 10 °C min^{-1} in a He flow (50 mL min^{-1}). A thermal conductivity detector (TCD) was used to monitor desorbed NH_3. The amount of acid sites was determined by the fitting peak area of the profiles.

N_2O adsorption FTIR

The N_2O adsorption FTIR was carried out in the FTIR system by using a JASCO 4100 FTIR spectrometer equipped with a triglycine sulfate (TGS) detector. IR spectra of the clean disk were recorded in vacuo at 25 °C to obtain background spectrum. The sample was pressed into a self-supporting disk (20 mm diameter, 30−60 mg) and placed in an IR cell attached to a closed-gas circulation system. The sample was pretreated by evacuation at 500 °C for 1 h, followed by adsorption of 5−500 Pa N_2O at 25 °C.

XRD

XRD pattern was collected on a Rint-Ultima III (Rigaku) using a Cu Kα X-ray source (40 kV, 40 mA).

ICP-AES

Elemental analyses of samples were performed on an inductively coupled plasma-atomic emission spectrometer (ICP-AES, Shimadzu ICPE-9000).

AAS

The Na content of the products was determined using an atomic absorption spectrometer (AAS,

Shimadzu AA-6200).

FE-SEM

Field-emission scanning electron microscopic (FE-SEM) images of the powder samples were obtained on SU9000 (Hitachi) microscope operating at 1 kV.

N_2 adsorption and desorption

Nitrogen adsorption and desorption measurements to obtain information on the micro- and meso-porosities were conducted at -196 ºC on a Belsorp-mini II (MicrotracBEL).

UV-vis

UV-vis spectra were collected in the range of 190-900 nm on a V-650DS spectrometer (JASCO). The diffuse reflectance spectra were converted into the absorption spectra using the Kubelka-Munk function.

TG-DTA

The amount of carbon deposit in the used samples was determined by the weight loss from 250 to 800 ºC in a thermogravimetric (TG) profile, which was performed on a thermogravimetric-differential thermal analyzer (TG-DTA, Rigaku Thermo plus EVO II).

Catalytic tests

Direct oxidation of methane reaction

Online-reaction-analysis system was equipped with two six-port inlet valves (Supplementary Fig. 3). In a typical test, 100 mg of catalyst with a granular form (particle size 500−1000 μm) was charged into a quartz tube (inner diameter 4 mm), which was placed in an electric tube furnace. The catalyst was pretreated at 500 °C for 1 h in Ar flow. The reaction was conducted at 350 °C in a flowing gas mixture of CH_4, N_2O, H_2O, and Ar with flow rates of 10, 10, 2, and 3 $mL \cdot min^{-1}$. The outlet gas, containing the products, unreacted CH_4 and N_2O, was analyzed using two on-line gas chromatographs (GC; GC-2014, Shimadzu). One of the GCs was used with a Shin carbon ST 50/80 packed column (Agilent Technologies, inner diameter 3 mm, length 6 m) and a TCD detector. Specifically, GC-TCD was used to detect H_2, N_2O, CO, CO_2 and CH_4. The other GC was equipped with a Porapak Q 80/100 packed column (Agilent Technologies, inner diameter 3 mm, length 6 m), a flame ionization detector (FID), and a methanizer. The GC-FID was used to investigate CH_4, and the produced methanol (MeOH), dimethyl ether (DME), alkanes, and alkenes. The yield of each carbon-containing product was calculated by considering the number of carbon atoms. The methane conversion in this study was defined as the total obtained products, and calculated as:

$$C_{\mathrm{CH4}} = \frac{\sum(i * Ci)}{\sum(i * Ci) + CH4}$$

where C_{CH4} is the CH_4 conversion, i is the number of carbon atoms in product C_i, $\Sigma(i * C_i)$ is the total amount of carbon of all the products, and *CH4* is the amount of CH_4 detected at the same time.

The N_2O conversion was calculated as:

$$C_{N2O} = \frac{ni-na}{ni}$$

where C_{N2O} is the N_2O conversion, n_i is the initial N_2O molar weight, n_a is the N_2O molar weight after reaction.

Note that, the calculation methods of CH_4 and N_2O conversion were different, resulting in that the two values were not equal.

The product selectivity was calculated as:

$$S_{Ci} = \frac{i*Ci}{\Sigma(i*Ci)}$$

where S_{Ci} is the selectivity of the product C_i, $\Sigma(i*C_i)$ is the total amount of carbon of all the products.

The product yield was calculated as:

$$Y_{Ci} = \frac{i*Ci}{\Sigma(i*Ci)+CH4}$$

where Y_{Ci} is the yield of the product C_i, $\Sigma(i*C_i)$ is the total amount of carbon of all the products, and $CH4$ is the amount of CH_4 detected at the same time.

The product formation rates were calculated as:

$$r_{Ci} = Y_{Ci} * F_{CH4}/m_{cat}$$

where r_{Ci} is the formation rate of product C_i, F_{CH4} is the initial flow rate of CH_4, m_{cat} is the mass of catalyst. $r_{hydrocarbons} = 2*(r_{C2}^{=} + r_{C2}^{-}) + 3*(r_{C3}^{=} + r_{C3}^{-}) + 4*(r_{C4}^{=} + r_{C4}^{-}) + 5*(r_{C5}^{=} + r_{C5}^{-})$.

Methanol to hydrocarbons (MTH) reaction

The MTH reaction was performed using a fixed-bed reactor connected to an online gas-chromatograph (GC-2014, Shimadzu) equipped with a HP-PLOT/Q capillary column and a flame ionization detector. The 50/80 mesh zeolite pellets without a binder were placed in a 6 mm quartz tubular flow reactor. The pretreatment was conducted at 500 °C for 30 min under Ar (20 $mL \cdot min^{-1}$). After the pretreatment, the reactor was cooled to 350 °C and the MTH reaction was started. The pressure of methanol was set at 5 kPa with Ar gas as the carrier; the weight-to-feed ratio (W/F) for methanol was 68 $g \cdot h \cdot mol^{-1}$. The product stream was analyzed using a system that automatically injected the product into a gas chromatograph connected directly to the outlet of the reactor via a heated transfer line.

The conversion and selectivity were calculated as:

$$Conversion\ of\ methanol\ [\%] = 1 - \frac{\text{Amount of methanol (in reacted gas)}}{\text{Amount of detected compounds (in reacted gas)}} \times 100$$

$$Product\ selectivity\ [\%] = \frac{\text{Amount of the target product}}{\text{Amount of detected products (in reacted gas)}} \times 100$$

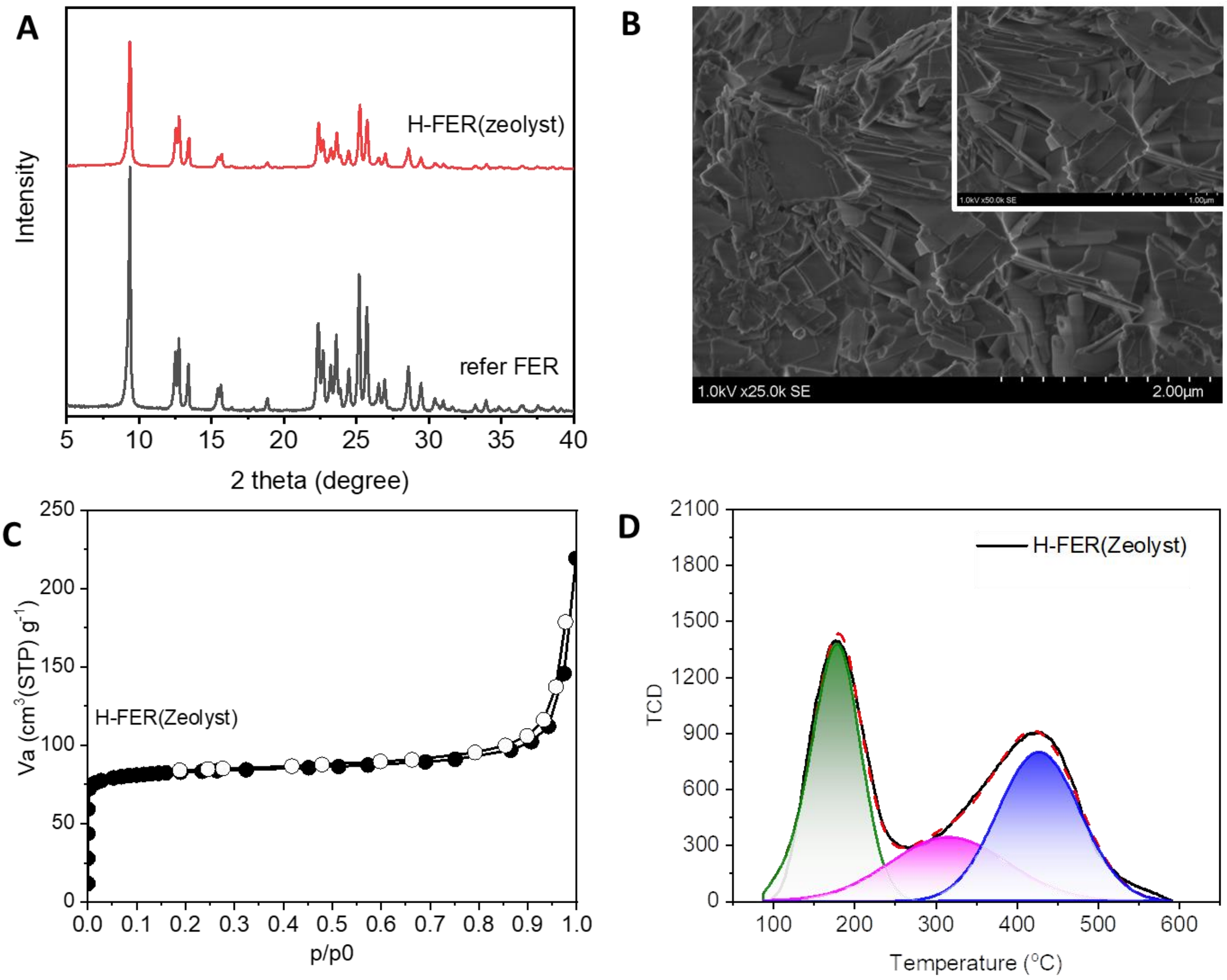

Figure S1. (A) XRD pattern, (B) SEM images, (C) N_2 adsorption and desorption pattern, (D) NH_3-TPD curve of H-FER zeolite (CP914C, Zeolyst).

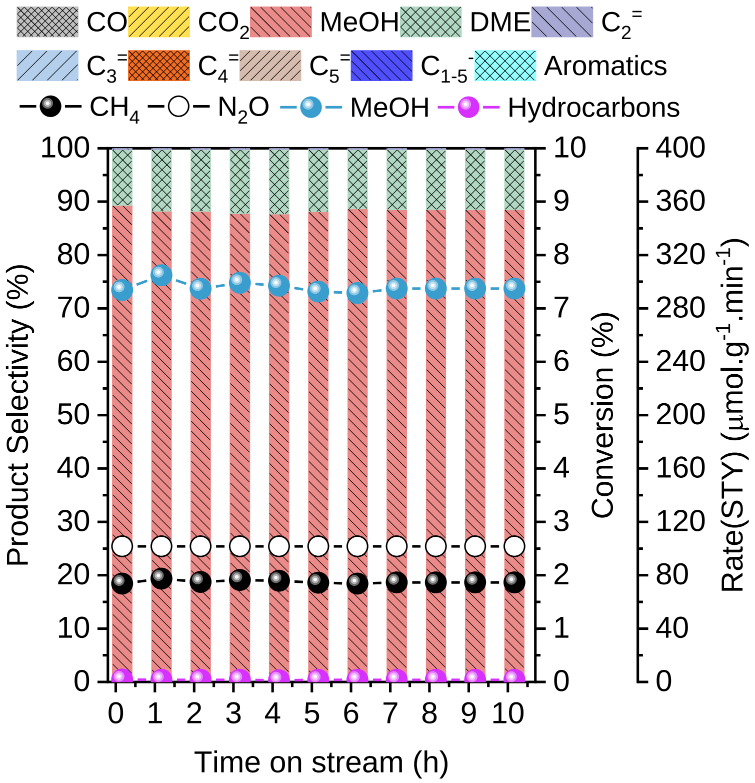

Figure S2. Stability test of H-FER-850 at 350 °C after activation at 500 °C for 1 h, reaction conditions: 25 mg catalyst, $CH_4/N_2O/H_2O/Ar$=10/10/5/0 ml·min^{-1}.

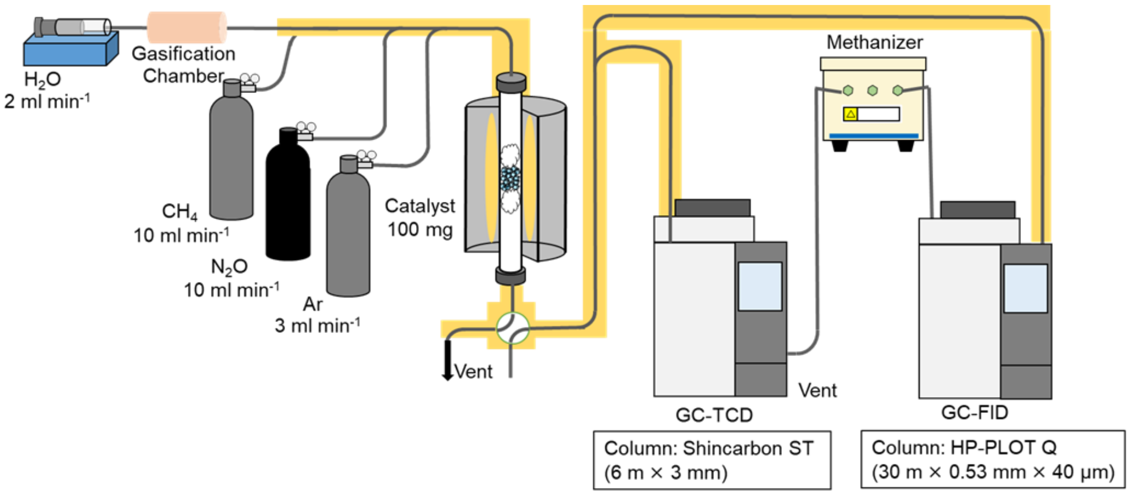

Figure S3. Fixed-bed flow reactor diagram for the direct oxidation of methane to methanol.

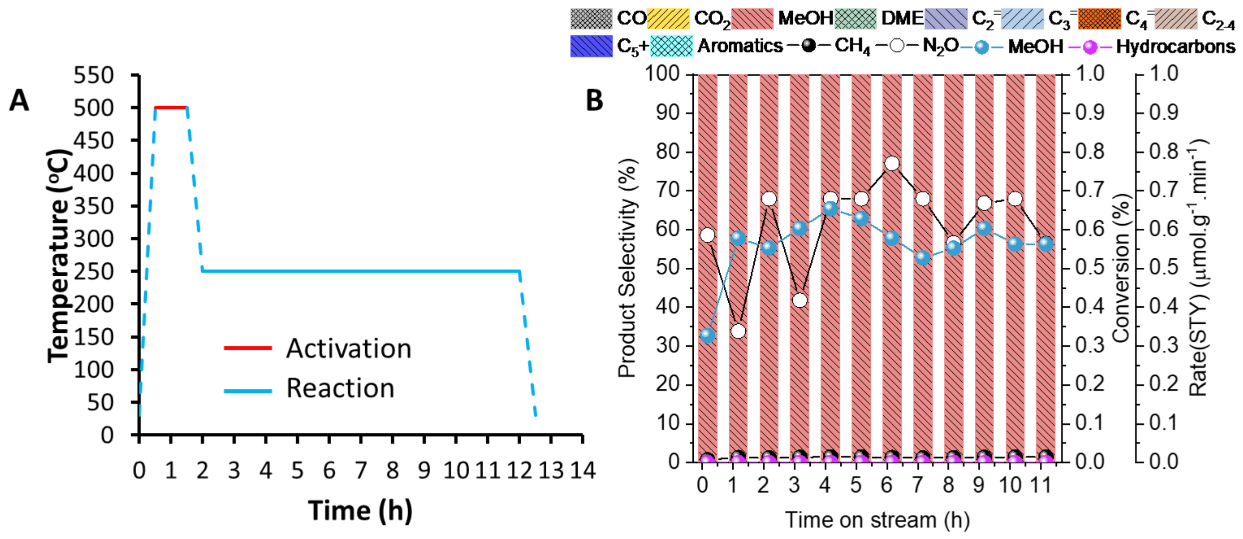

Figure S2. (A) Temperature program and (B) stability test in direct oxidation of methane to methanol at 250 °C. Reaction conditions: 100 mg H-FER as catalyst, $CH_4/N_2O/H_2O/Ar$ = 10/10/2/3 $ml\cdot min^{-1}$, WHSV =15000 $ml\cdot g^{-1}\cdot h^{-1}$.

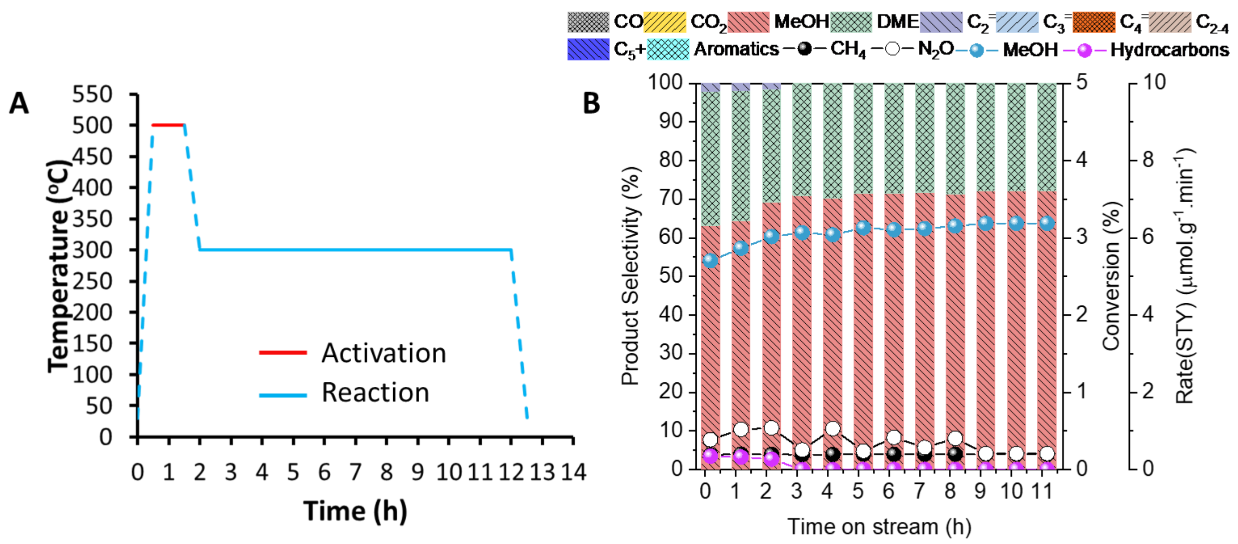

Figure S3. (A) Temperature program and (B) stability test in direct oxidation of methane to methanol at 300 ºC. Reaction conditions: 100 mg H-FER as catalyst, $CH_4/N_2O/H_2O/Ar$ = 10/10/2/3 ml·min^{-1}, WHSV =15000 ml·g^{-1}·h^{-1}.

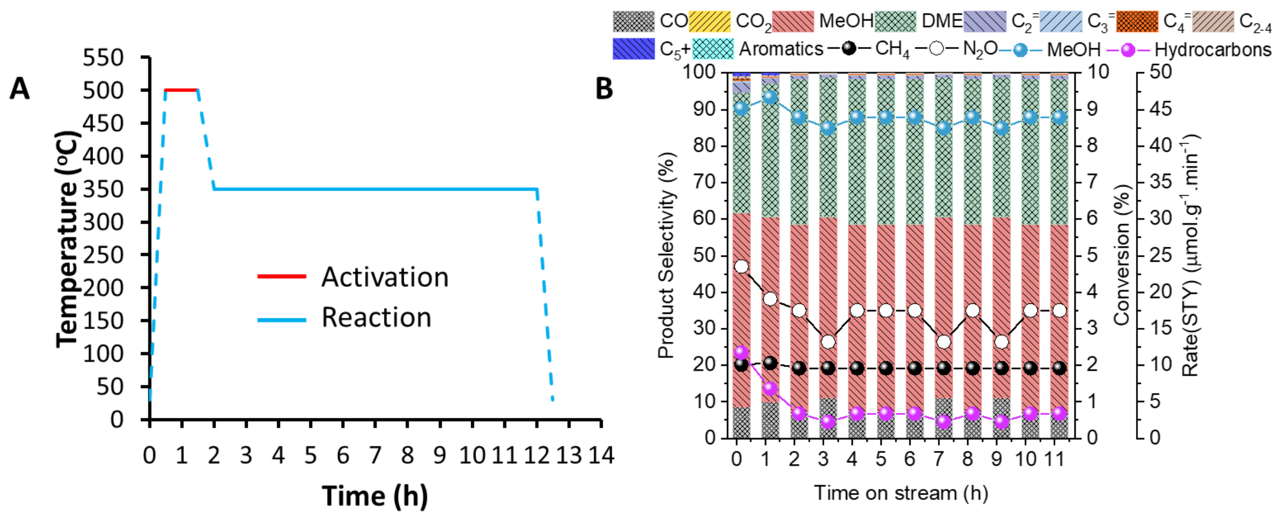

Figure S4. (A) Temperature program and (B) stability test in direct oxidation of methane to methanol at 350 ºC. Reaction conditions: 100 mg H-FER as catalyst, $CH_4/N_2O/H_2O/Ar$ = 10/10/2/3 ml·min^{-1}, WHSV =15000 ml·g^{-1}·h^{-1}.

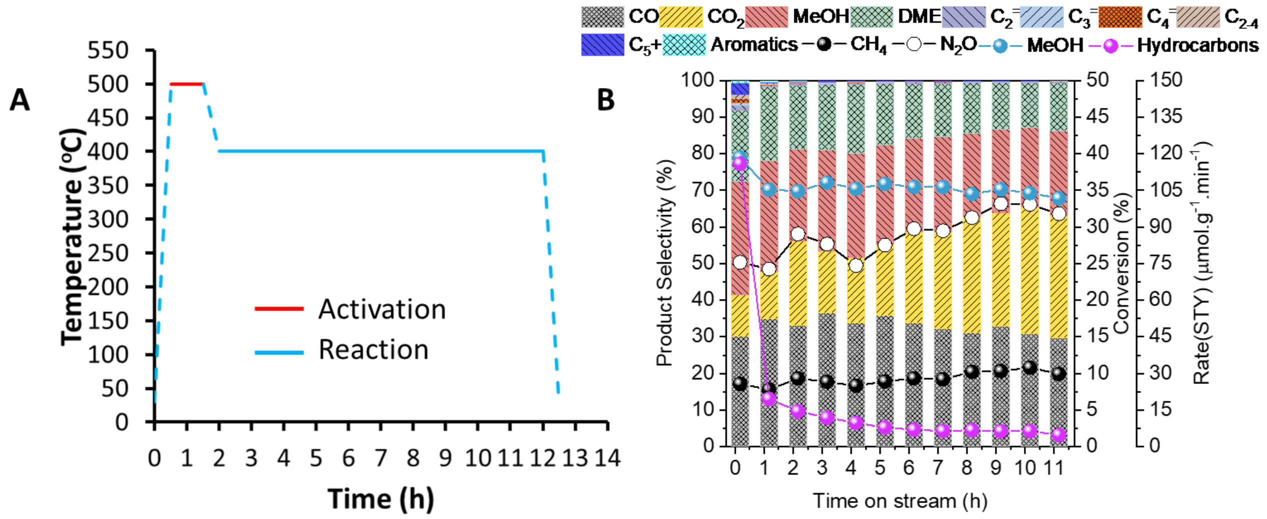

Figure S5. (A) Temperature program and (B) stability test in direct oxidation of methane to methanol at 400 ºC. Reaction conditions: 100 mg H-FER as catalyst, $CH_4/N_2O/H_2O/Ar$ = 10/10/2/3 ml·min^{-1}, WHSV =15000 ml·g^{-1}·h^{-1}.

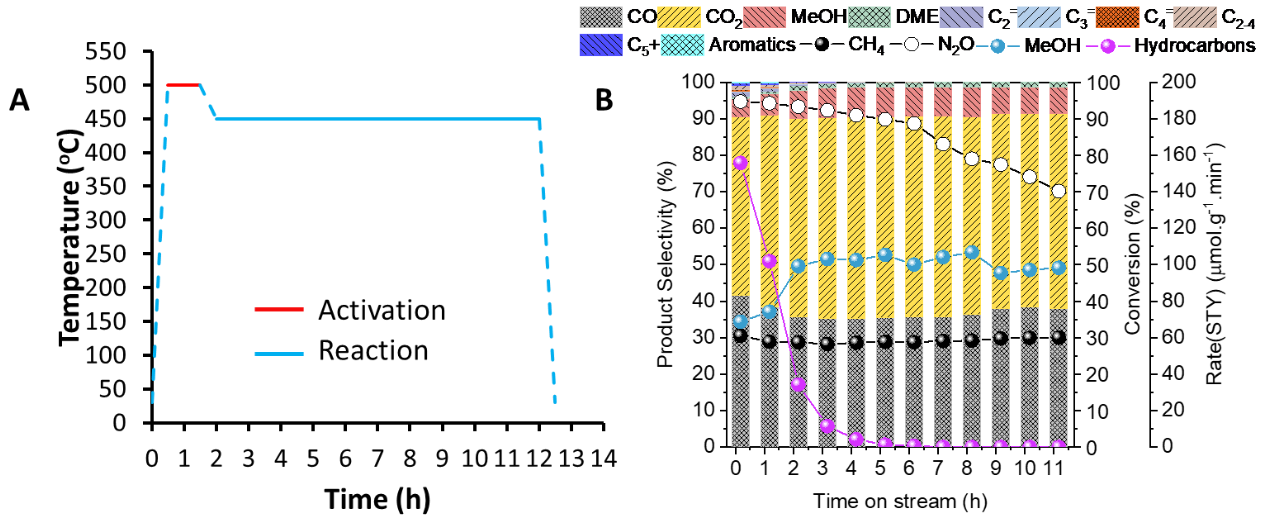

Figure S6. (A) Temperature program and (B) stability test in direct oxidation of methane to methanol at 450 ºC. Reaction conditions: 100 mg H-FER as catalyst, $CH_4/N_2O/H_2O/Ar$ = 10/10/2/3 $ml\cdot min^{-1}$, WHSV =15000 $ml\cdot g^{-1}\cdot h^{-1}$.

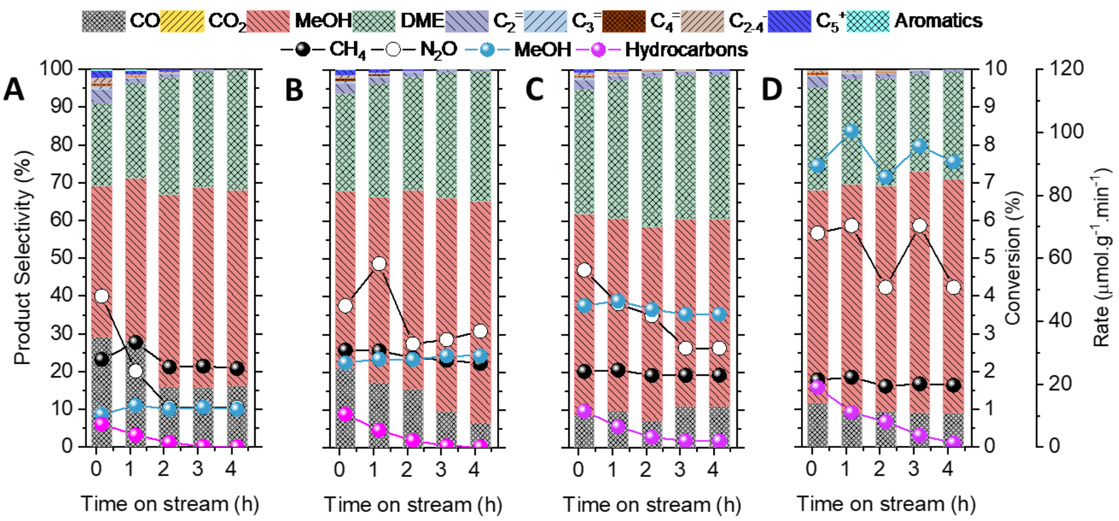

Figure S9. Effects of reactant flow rate (A) $CH_4/N_2O/H_2O/Ar$ = 2.5/2.5/0.5/0.75 ml·min^{-1} (B) $CH_4/N_2O/H_2O/Ar$ = 5/5/1/1.5 ml·min^{-1}, (C) $CH_4/N_2O/H_2O/Ar$ = 10/10/2/3 ml·min^{-1}, and (D) $CH_4/N_2O/H_2O/Ar$ = 20/20/4/6 ml min^{-1} on the reaction performance in direct oxidation of methane to methanol. Reaction conditions: 100 mg H-FER as catalyst, 350 ºC reaction after activation at 500 ºC for 1 h.

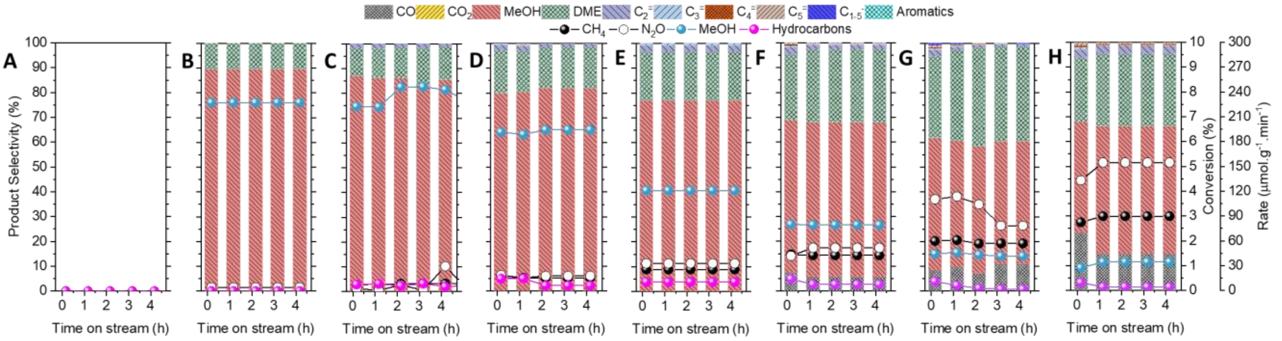

Figure S10. Effects of catalyst amount (A) 0, (B) 2.5, (C) 5, (D) 10, (E) 25, (F) 50, (G) 100, (H) 200 mg on the reaction performance in direct oxidation of methane to methanol. Reaction conditions: H-FER as catalyst, 350 ºC reaction after activation at 500 ºC for 1 h, $CH_4/N_2O/H_2O/Ar$ = 10/10/2/3 ml·min^{-1}.

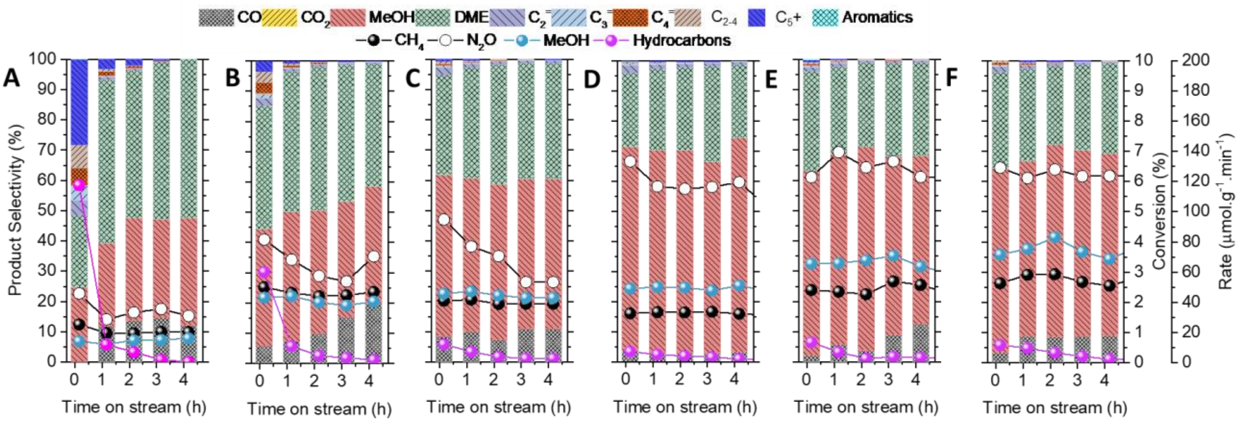

Figure S7. Effects of H_2O flow rate in the reactant on the reaction performance of direct oxidation of methane to methanol (A) 0, (B) 1, (C) 2, (D) 3, (E) 4, and (F) 5 ml ml·min^{-1} H_2O flow rate. Reaction conditions: 100 mg H-FER as catalyst, 350 ºC reaction after activation at 500 ºC for 1 h, $CH_4/N_2O/H_2O/Ar$ = 10/10/x/(5-x) ml·min^{-1}, x=0-5.

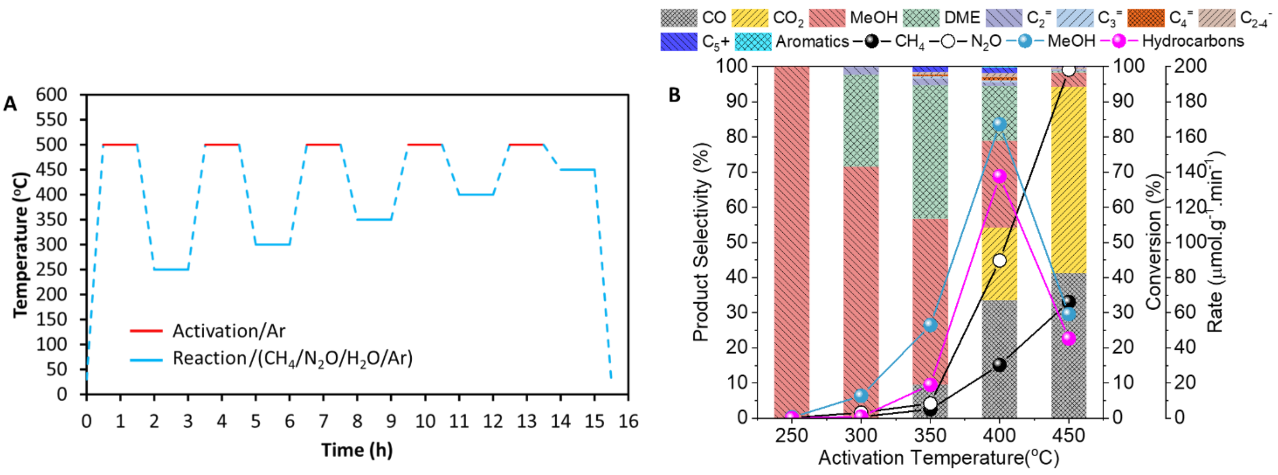

Figure S82. (A) Temperature program of the same catalyst continuous reaction at 250-450 ºC after activation at 500 ºC for 1 h. (B) Reaction performance of H-FER continuous reaction at 250-450 ºC after activation at 500 ºC for 1 h.

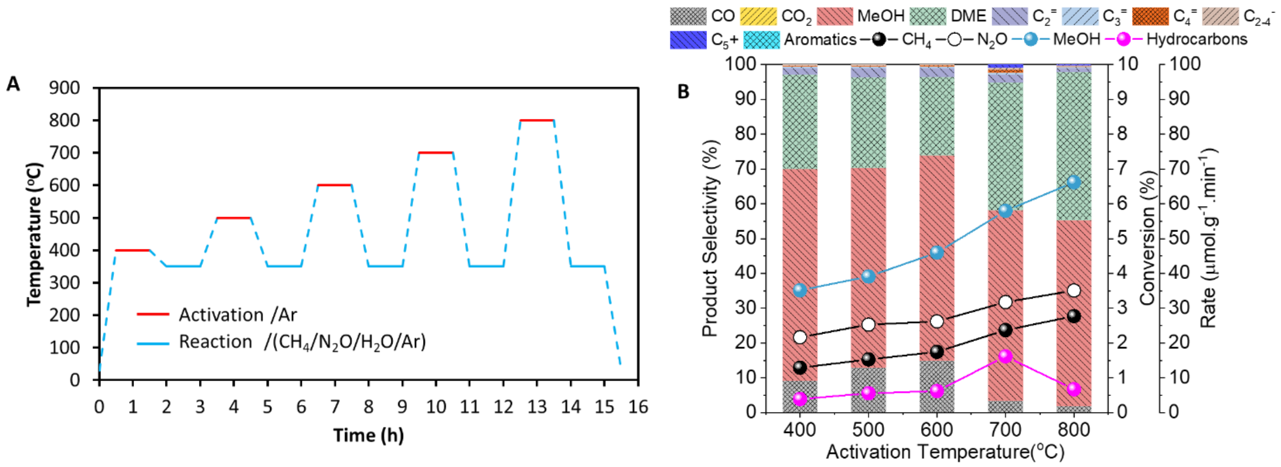

Figure S93. (A) Temperature program of the same catalyst continuous reaction at 350 ºC after activation at 400-800 ºC for 1 h. (B) Reaction performance of H-FER continuous reaction at 350 ºC after activation at 400-800 ºC for 1 h.

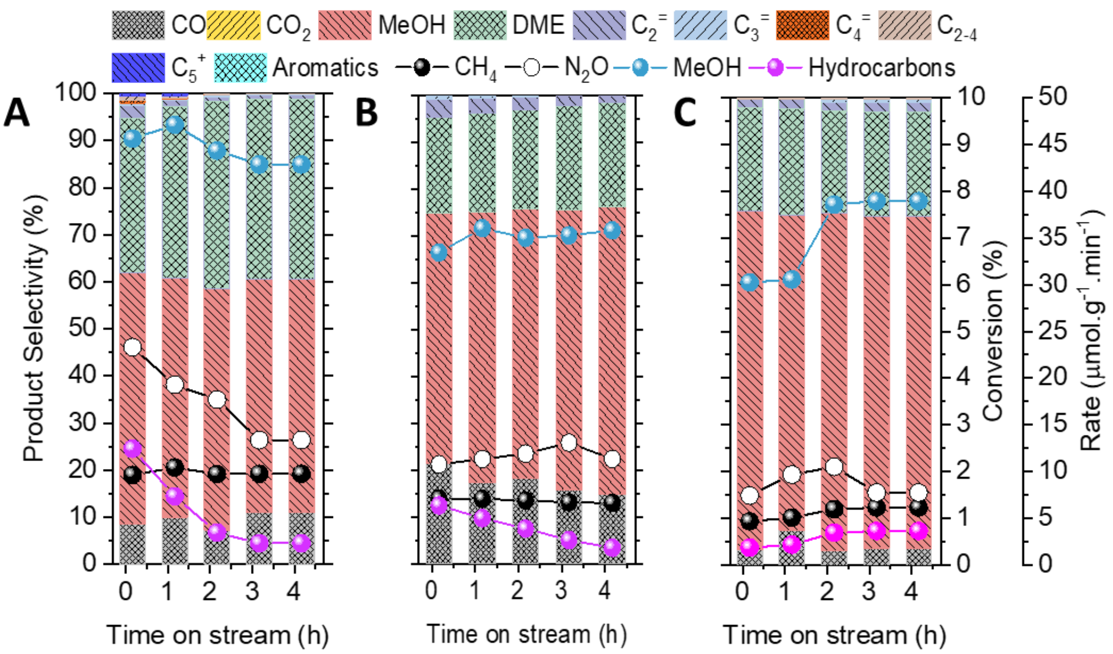

Figure S104. Stability test of (A) H-FER, (B) Na/FER, and (C) K/FER zeolite in direct oxidation of methane to methanol. Reaction conditions: 100 mg catalyst, 350 ºC, $CH_4/N_2O/H_2O/Ar$ = 10/10/2/3 $ml\cdot min^{-1}$, WHSV =15000 $ml\cdot g^{-1}\cdot h^{-1}$.

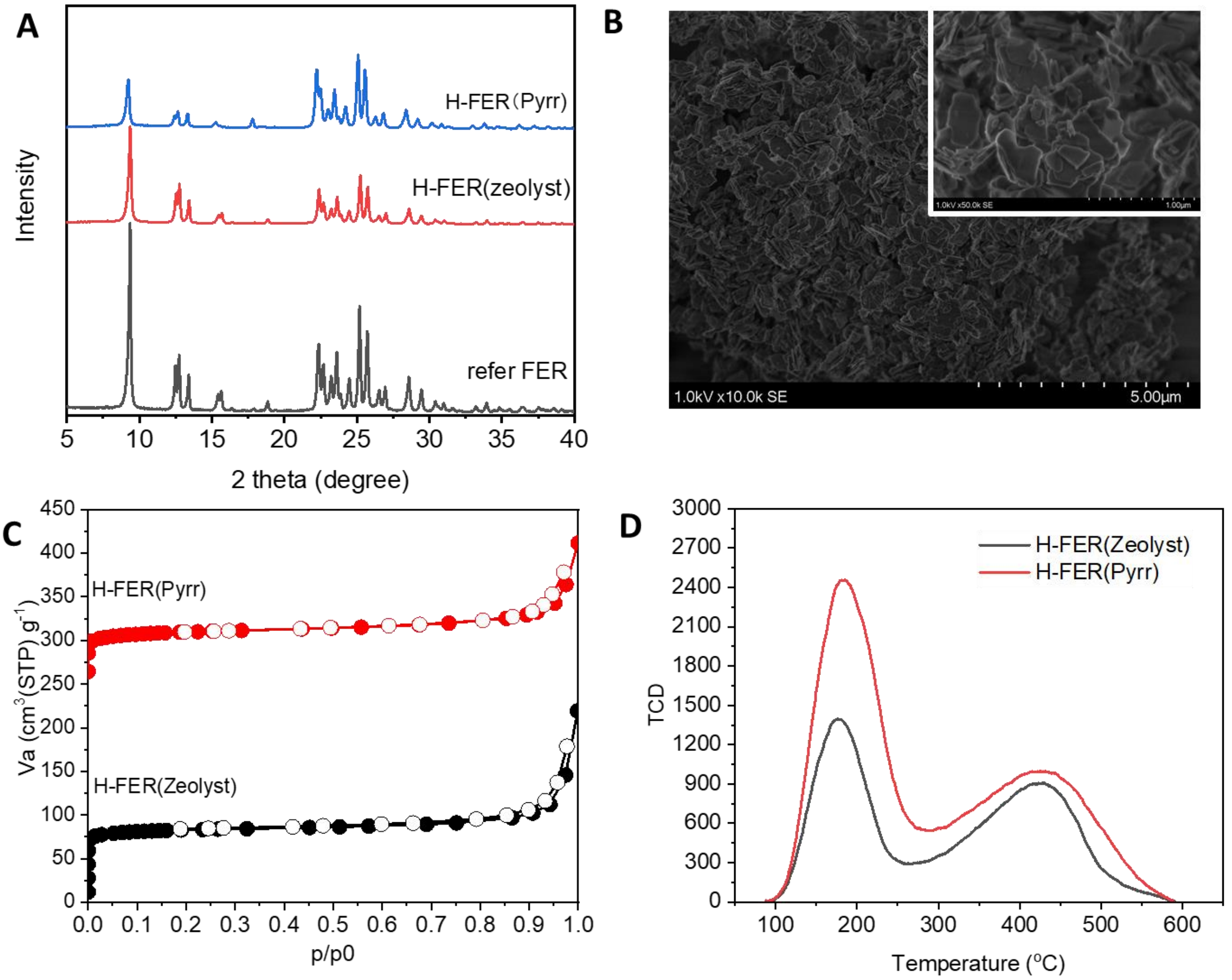

Figure S15. Comparison (A) XRD patterns, (B) SEM image, (C) N_2 adsorption and desorption patterns, and (D) NH_3-TPD curves of H-FER(Pyrr) zeolite with H-FER (CP914C, Zeolyst).

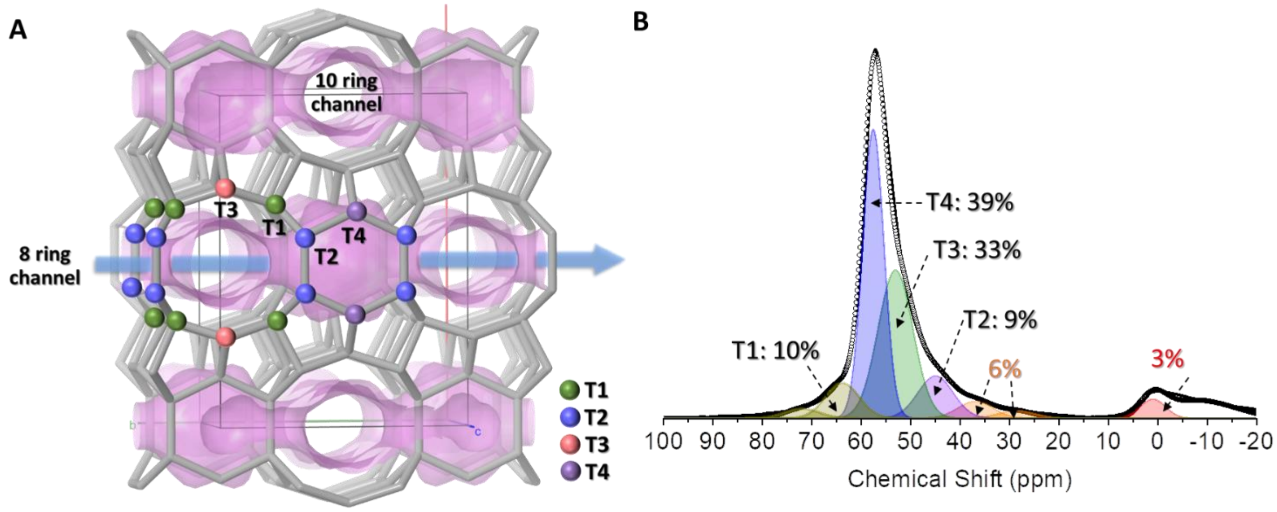

Figure S16. (A) The positions of the four crystallographically independent T sites, and the two perpendicular intersecting 8 and 10 ring channels. (B) Deconvolution of ^{27}Al MAS NMR spectra and the proportion at corresponding T site for H-FER (Pyrr) zeolite.

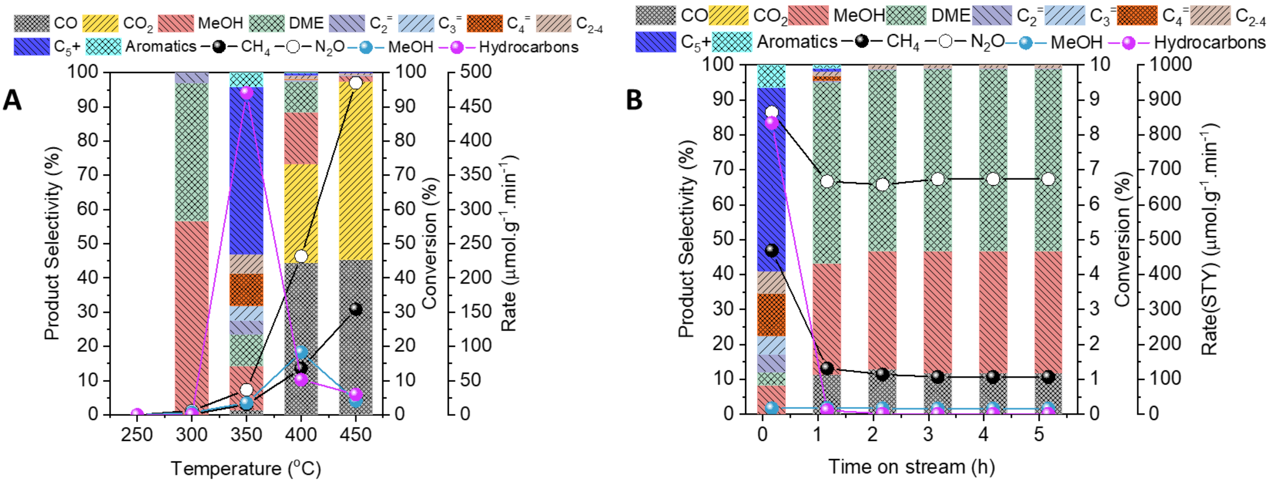

Figure S17. (A) Catalytic performance of H-FER(Pyrr) at 250-450 ºC after activation at 500 ºC for 1 h, reaction conditions: 100 mg catalyst, $CH_4/N_2O/H_2O/Ar$=10/10/2/3 ml·min^{-1}, WHSV=15000 ml·g^{-1}·h^{-1}. (B) Stability test of H-FER(Pyrr) at 350 ºC after activation at 500 ºC for 1 h, reaction conditions: 100 mg catalyst, $CH_4/N_2O/H_2O/Ar$=10/10/2/3 ml·min^{-1}, WHSV=15000 ml·g^{-1}·h^{-1}.

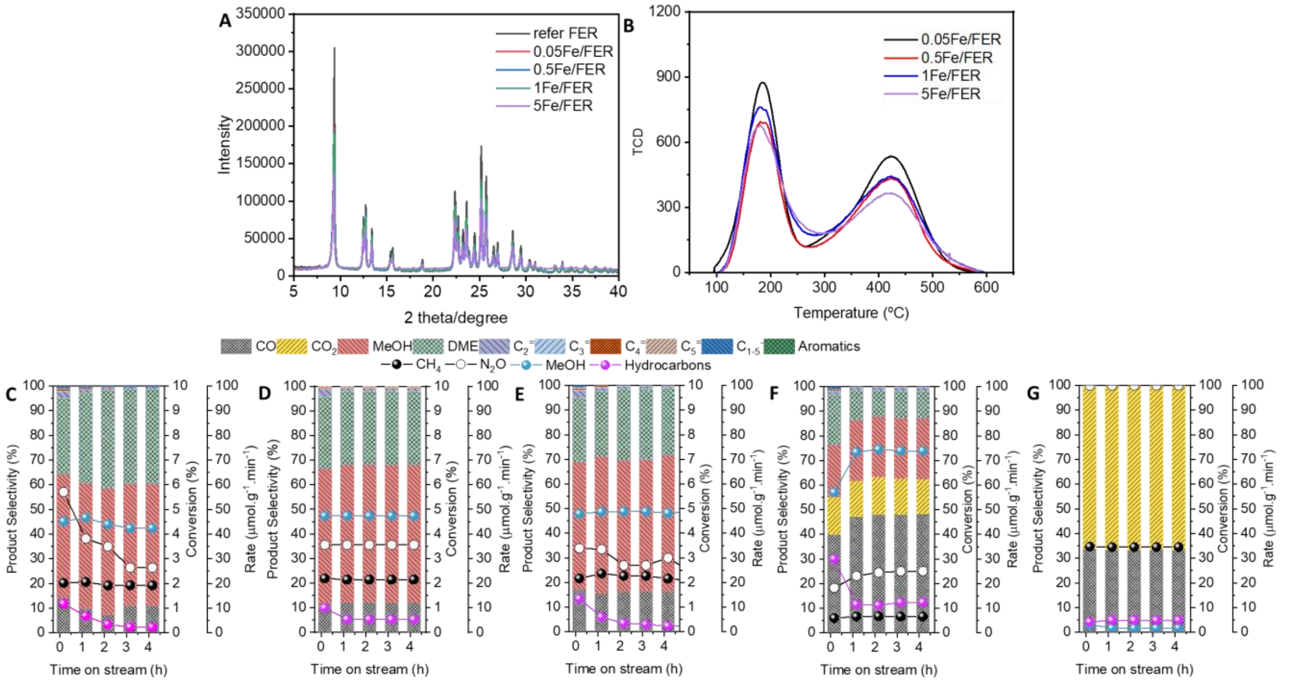

Figure S118. (A) XRD patterns and (B) NH_3-TPD curves of *x*Fe/FER zeolites. Reaction performance of (B) H-FER, (C) 0.05Fe/FER, (D) 0.5Fe/FER, (D) 1Fe/FER, and (F) 5Fe/FER zeolites. Reaction conditions: 100 mg catalyst, 350 °C, $CH_4/N_2O/H_2O/Ar$ = 10/10/2/3 ml·min^{-1}, WHSV =15000 ml·g^{-1}·h^{-1}.

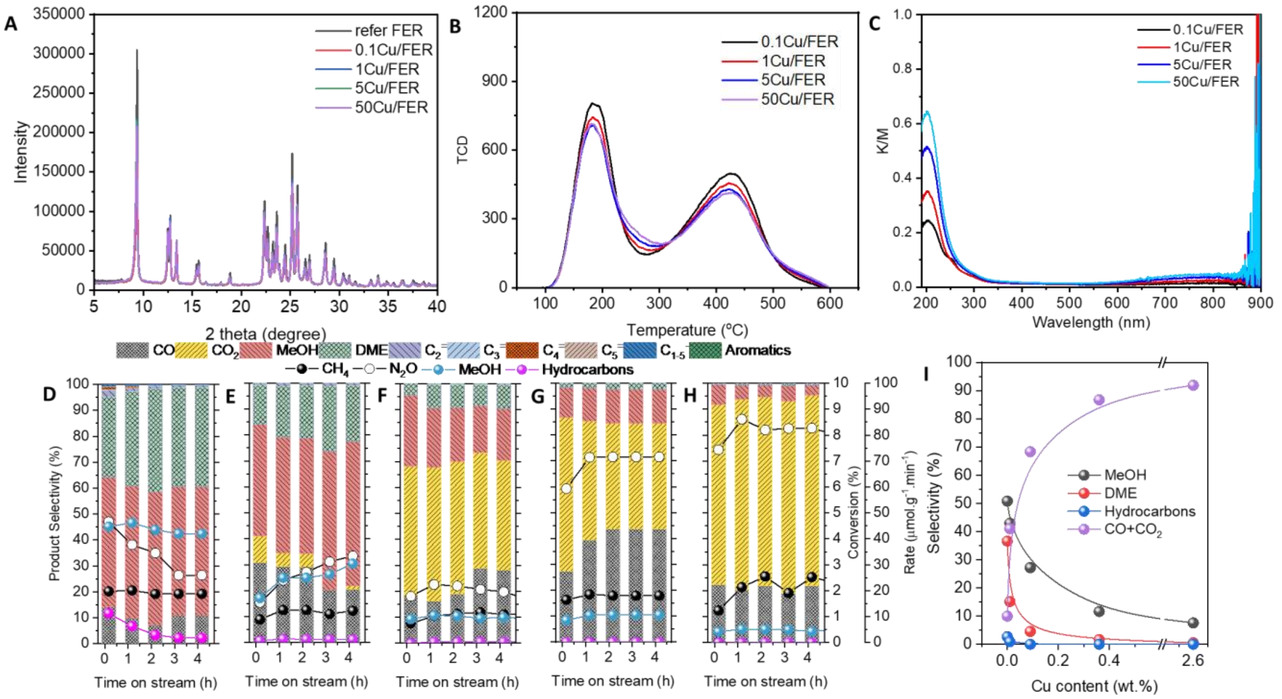

Figure S129. (A) XRD patterns, (B) NH_3-TPD curves, (C) UV-vis spectra of *y*Cu/FER zeolites. Reaction performance of (D) H-FER, (E) 0.1Cu/FER, (F) 1Cu/FER, (G) 5Cu/FER, and (H) 50Cu/FER zeolites. Reaction conditions: 100 mg catalyst, 350 ºC, $CH_4/N_2O/H_2O/Ar$ = 10/10/2/3 ml·min^{-1}, WHSV =15000 ml·g^{-1}·h^{-1}. (I) Selectivity as a function of Cu content of *y*Cu/FER zeolites at TOS=0.17 h. Reaction conditions: 100 mg catalyst, 350 ºC, $CH_4/N_2O/H_2O/Ar$ = 10/10/2/3 ml·min^{-1}.

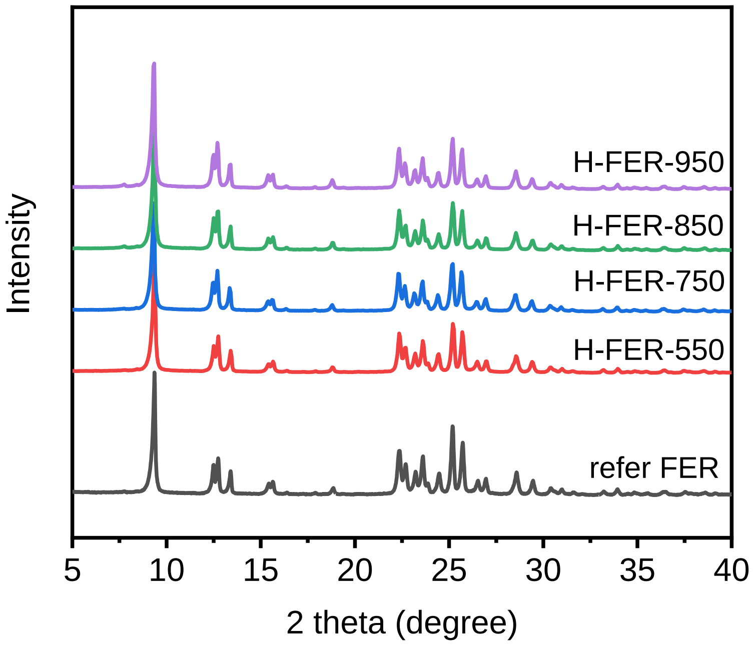

Figure S20. XRD patterns of H-FER-*t* zeolites (CP914C, Zeolyst).

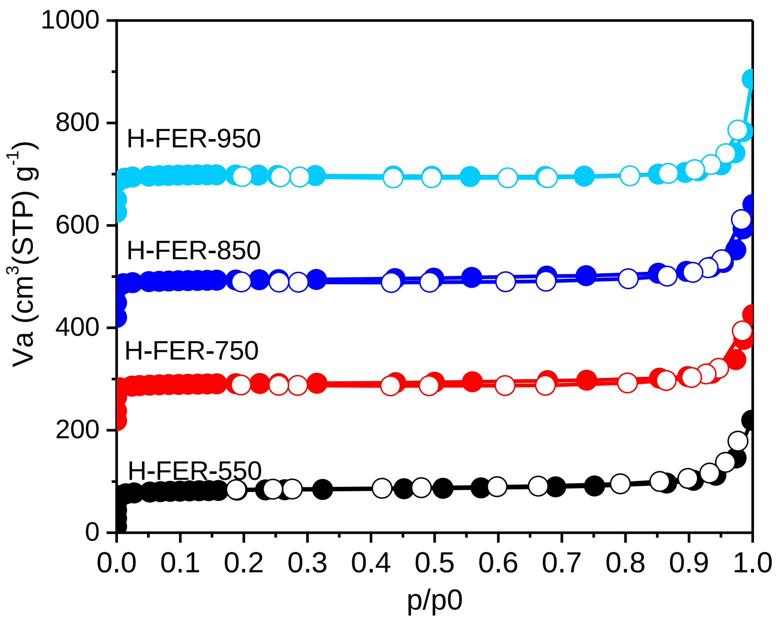

Figure S21. N_2 adsorption and desorption curves of H-FER-*t* zeolites (CP914C, Zeolyst).

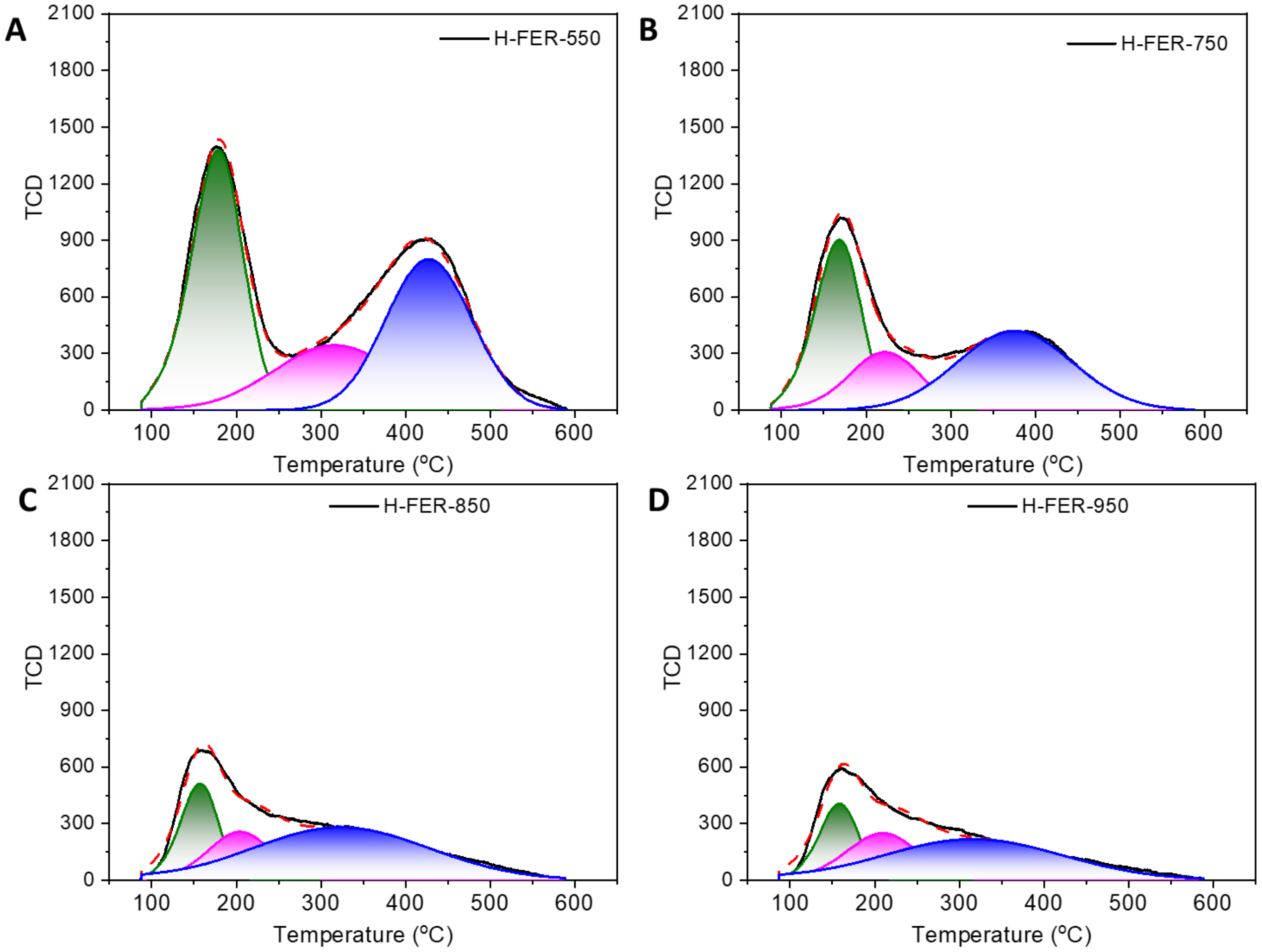

Figure S22. Deconvolution of NH_3-TPD curves of (A) H-FER-550, (B) H-FER-750, (C) H-FER-850, (D) H-FER-950 zeolites.

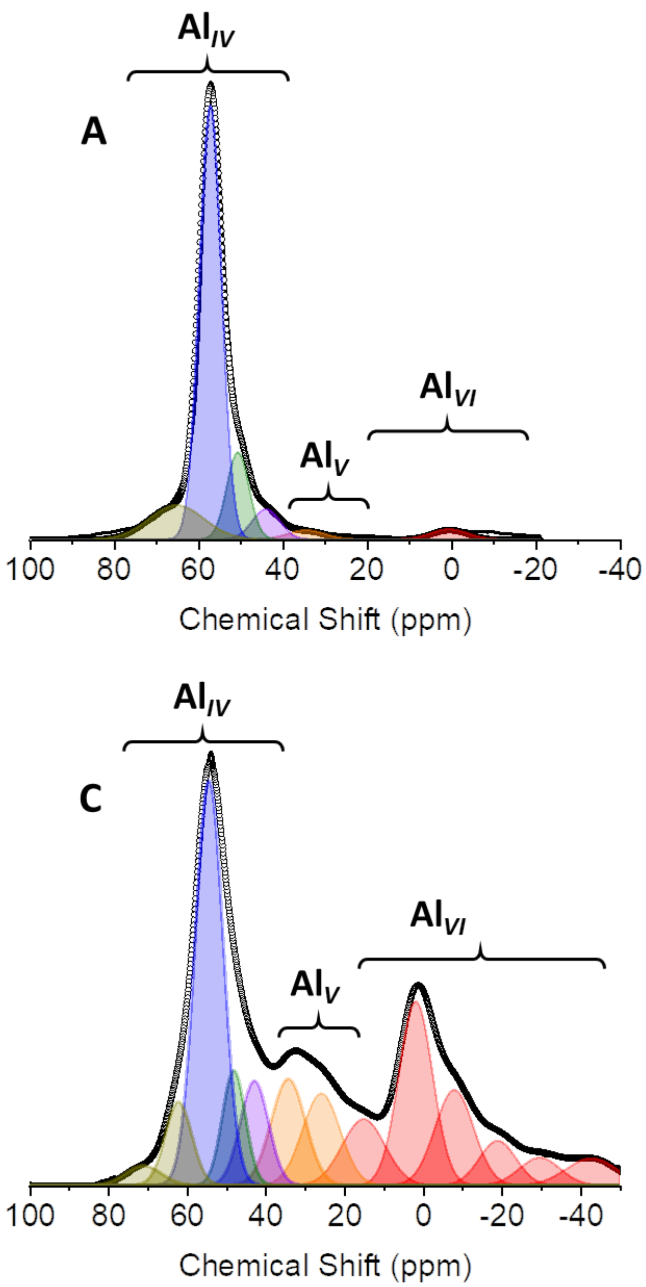

Figure S23. Deconvolution of ^{27}Al MAS NMR spectra of (A) H-FER-550, (B) H-FER-750, (C) H-FER-850, (D) H-FER-950 zeolites.

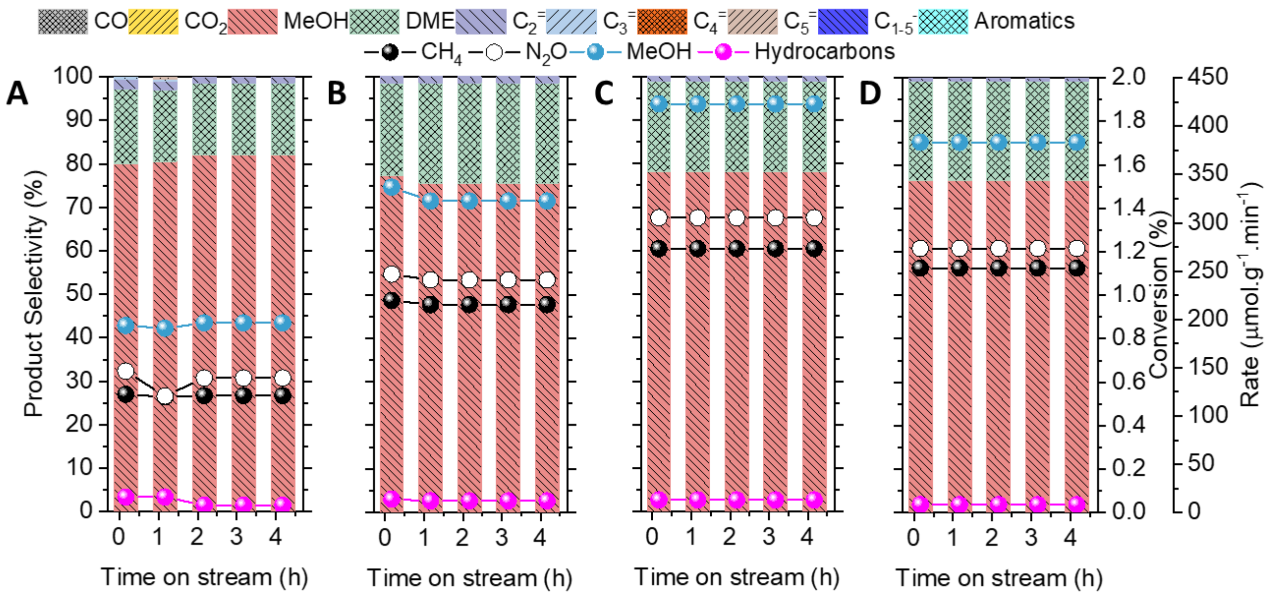

Figure S24. Reaction performance of FER after calcination at (A) 550 ºC, (B) 750 ºC, (C) 850 ºC, (D) 950 ºC. Reaction conditions: 10 mg catalyst, 350 ºC, $CH_4/N_2O/H_2O/Ar$ = 10/10/2/3 ml·min^{-1}, WHSV =15000 ml·g^{-1}·h^{-1}.

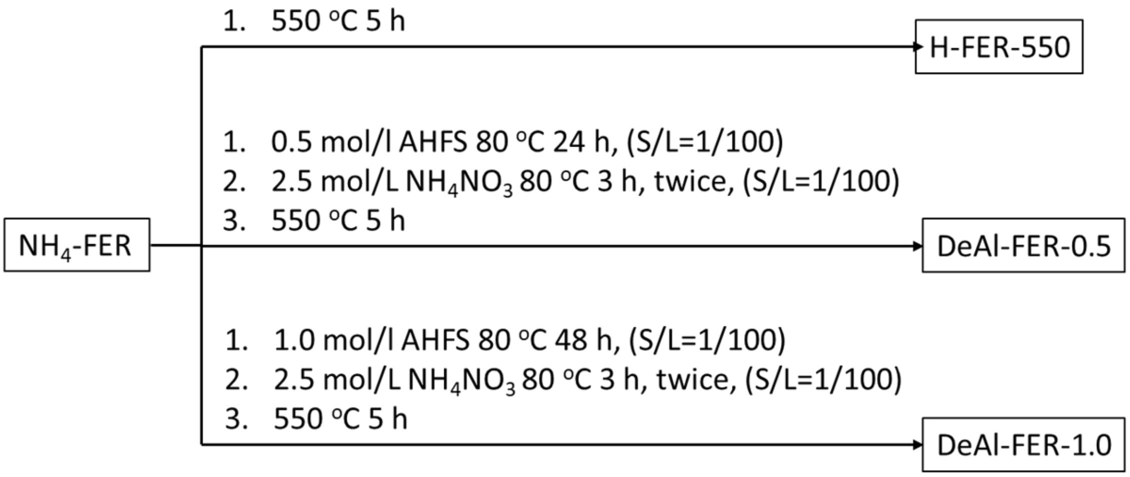

Figure S25. The preparation process of dealumination for FER zeolite.

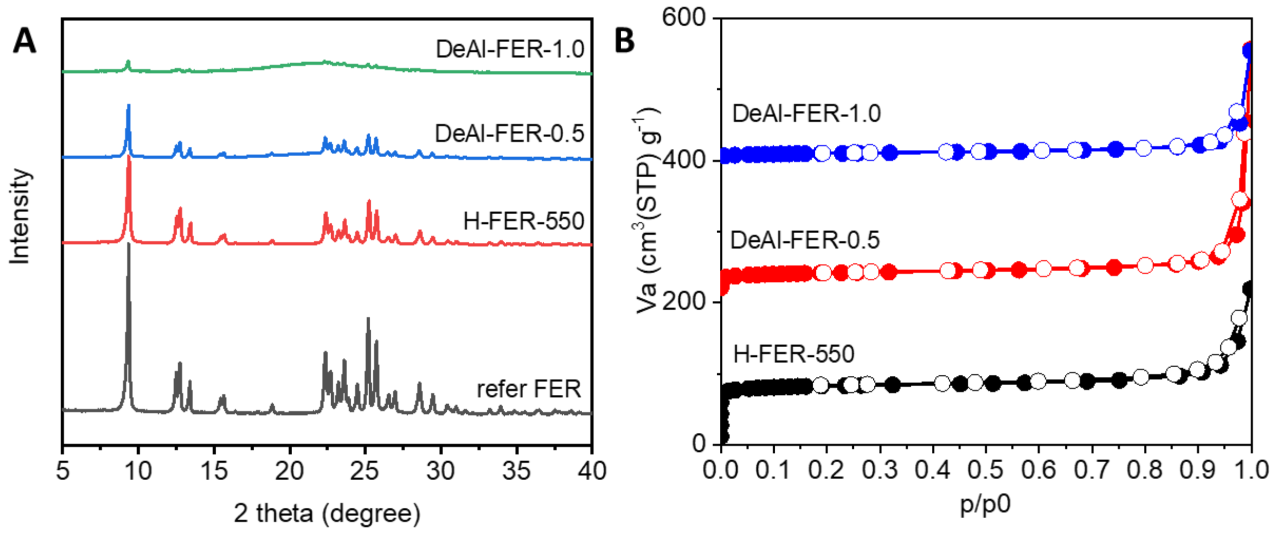

Figure S26. (A) XRD patterns and (B) N_2 adsorption and desorption patterns of the dealuminated FER zeolites.

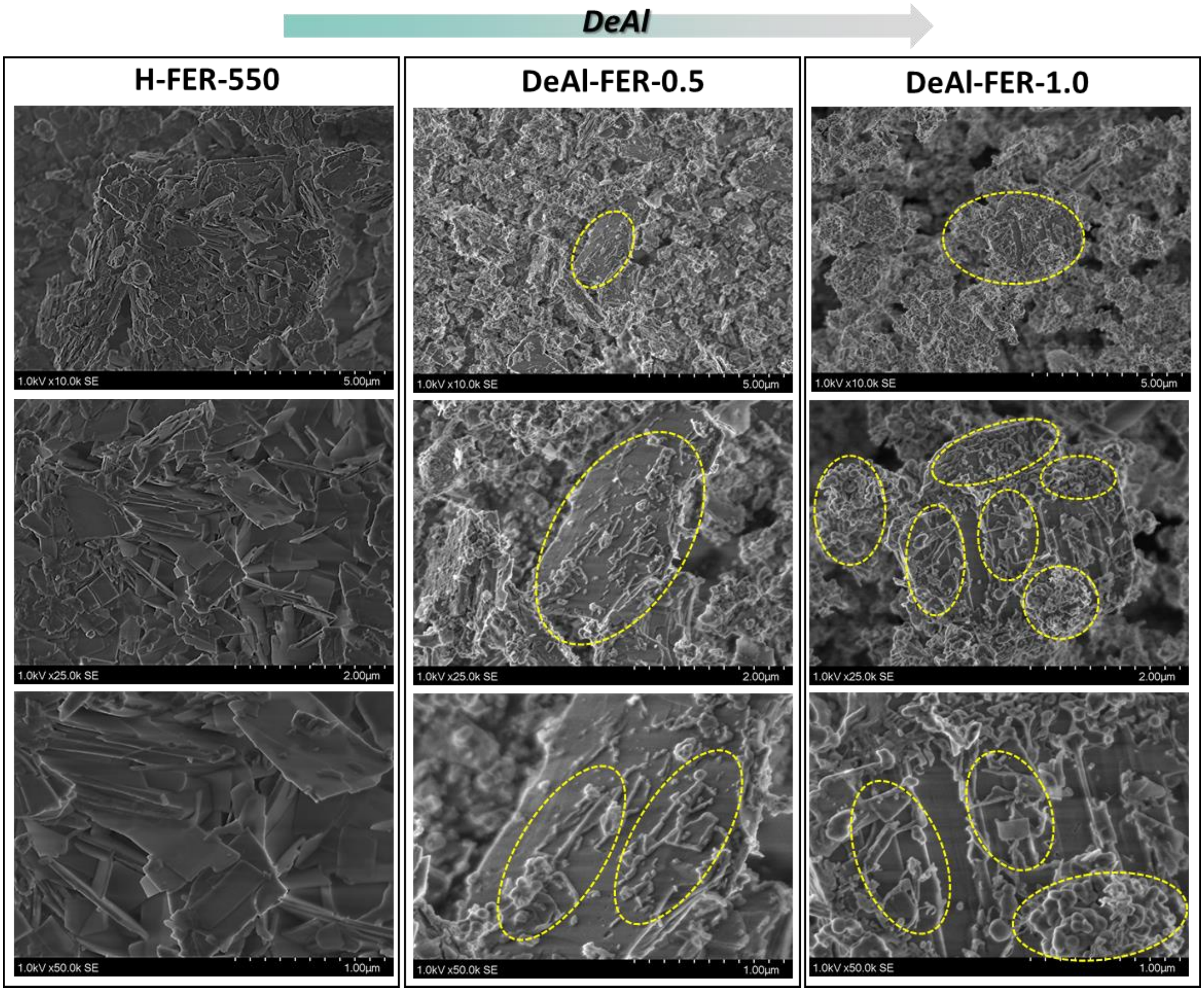

Figure S27. SEM images of the dealuminated FER zeolites.

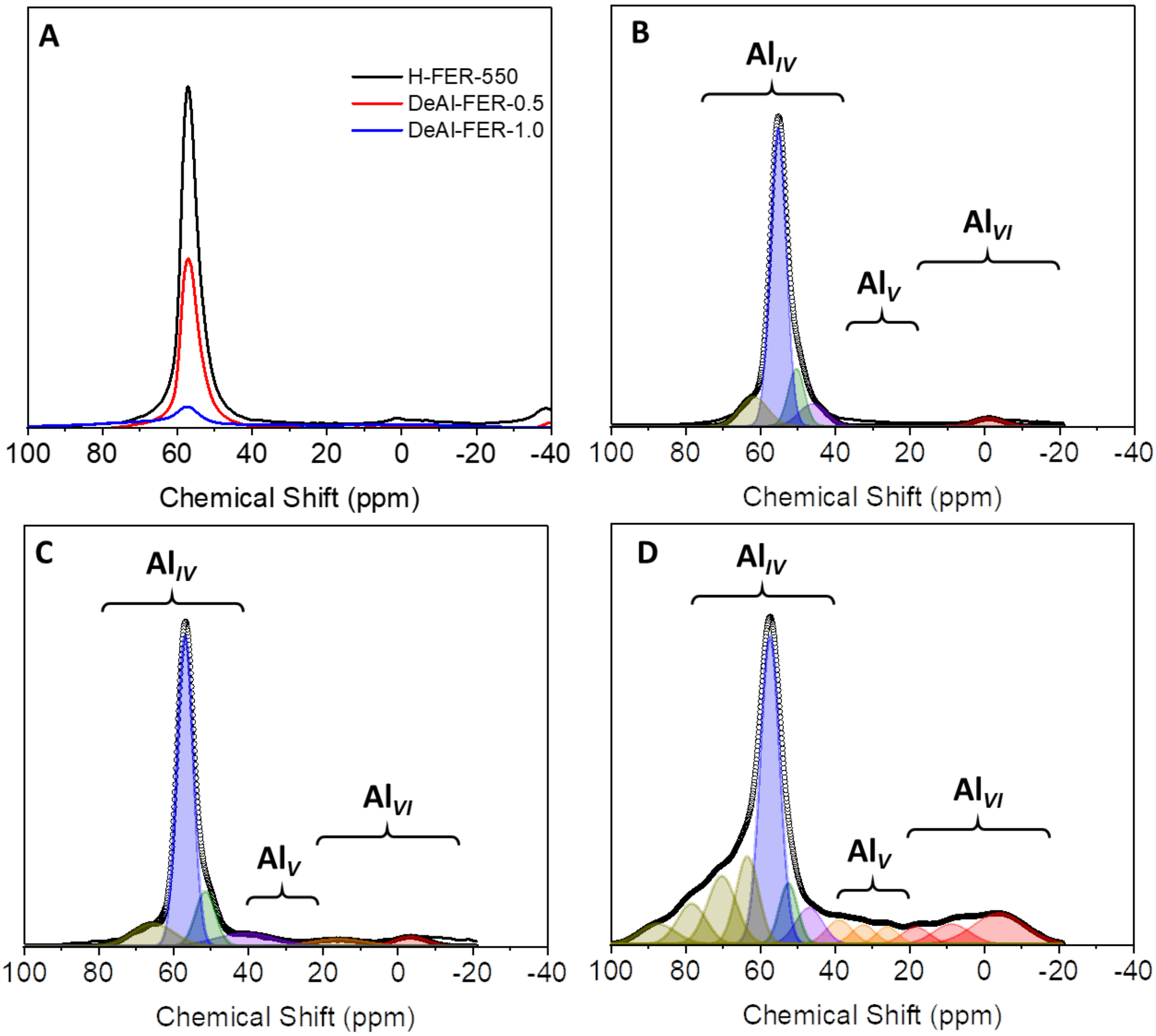

Figure S28. (A) Compare the ^{27}Al MAS NMR spectra of H-FER-550, DeAl-FER-0.5, DeAl-FER-1.0. Deconvolution of ^{27}Al MAS NMR spectra of (B) H-FER-550, (C) DeAl-FER-0.5, and (D) DeAl-FER-1.0 zeolites.

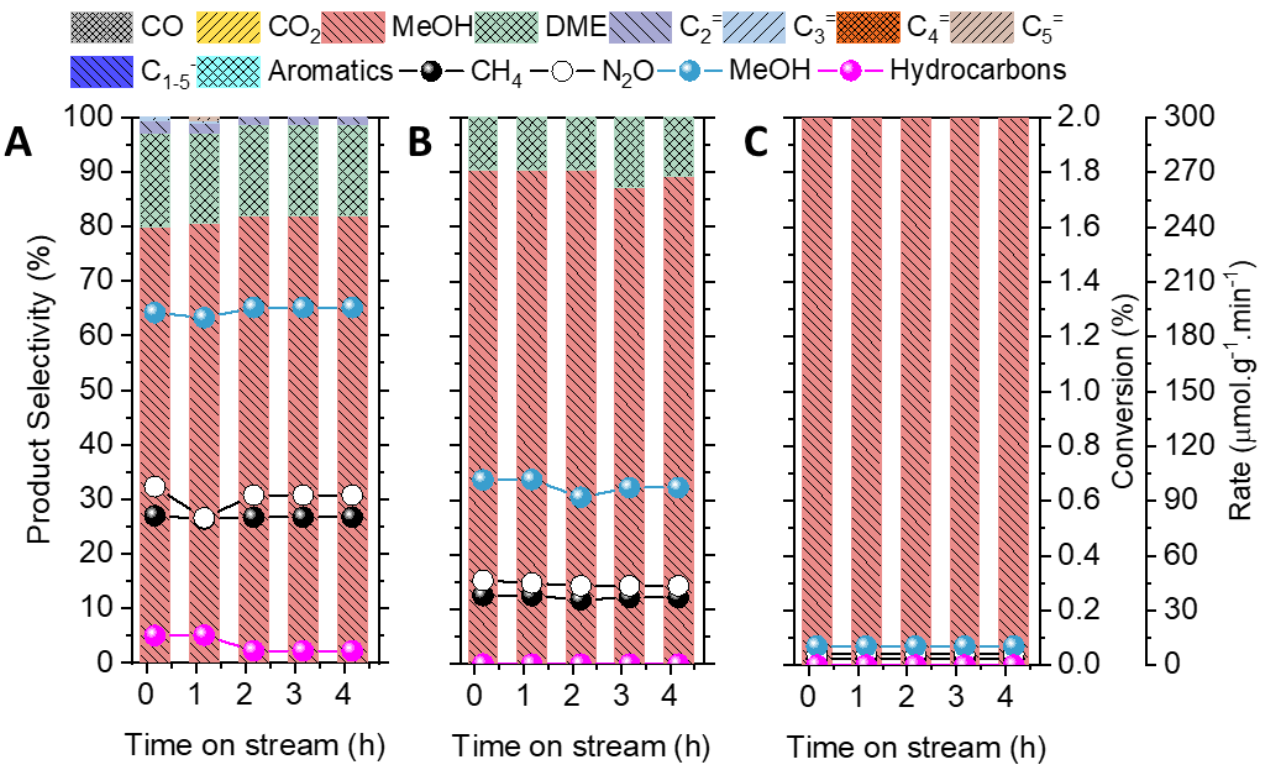

Figure S29. Stability test of (A) H-FER, (B) DeAl-FER-0.5, and (C) DeAl-FER-1.0 zeolite in direct oxidation of methane to methanol. Reaction conditions: 10 mg catalyst, 350 ºC, $CH_4/N_2O/H_2O/Ar$ = 10/10/2/3 ml·min^{-1}, WHSV =15000 ml·g^{-1}·h^{-1}.

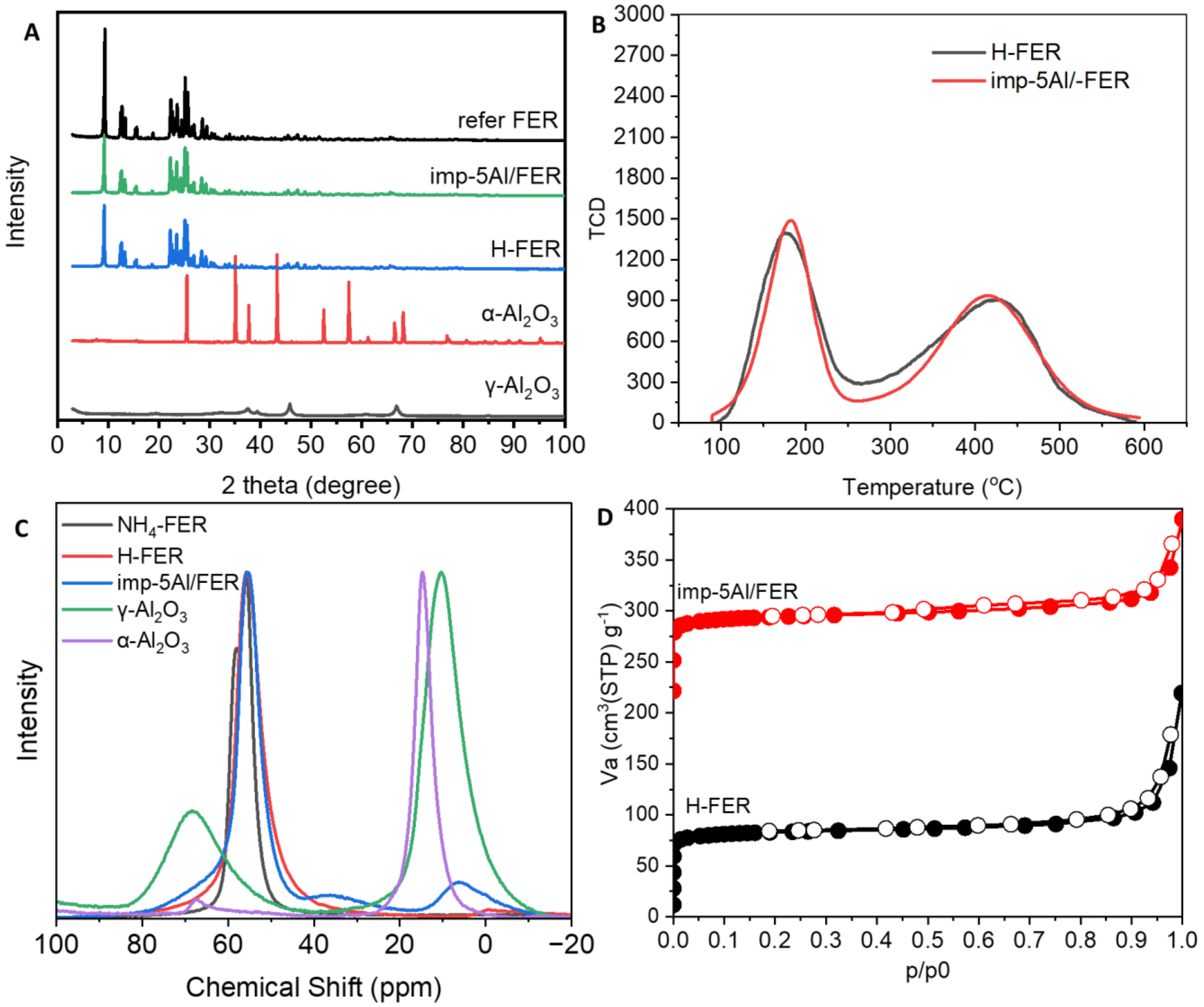

Figure S30. (A) XRD patterns, (B) NH_3-TPD curves, (C) ^{27}Al MAS NMR spectra, and (D) N_2 adsorption and desorption curves of FER zeolites after impregnation Al.

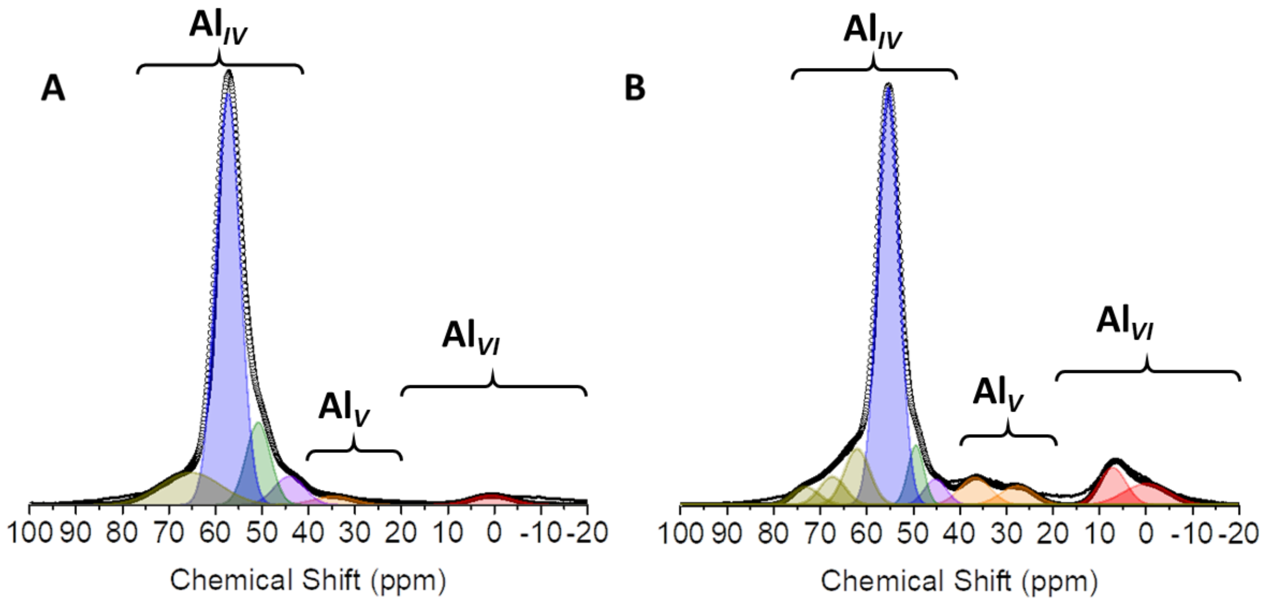

Figure S31. Deconvolution of ^{27}Al MAS NMR spectra of (A) H-FER and (B) imp-5Al/FER.

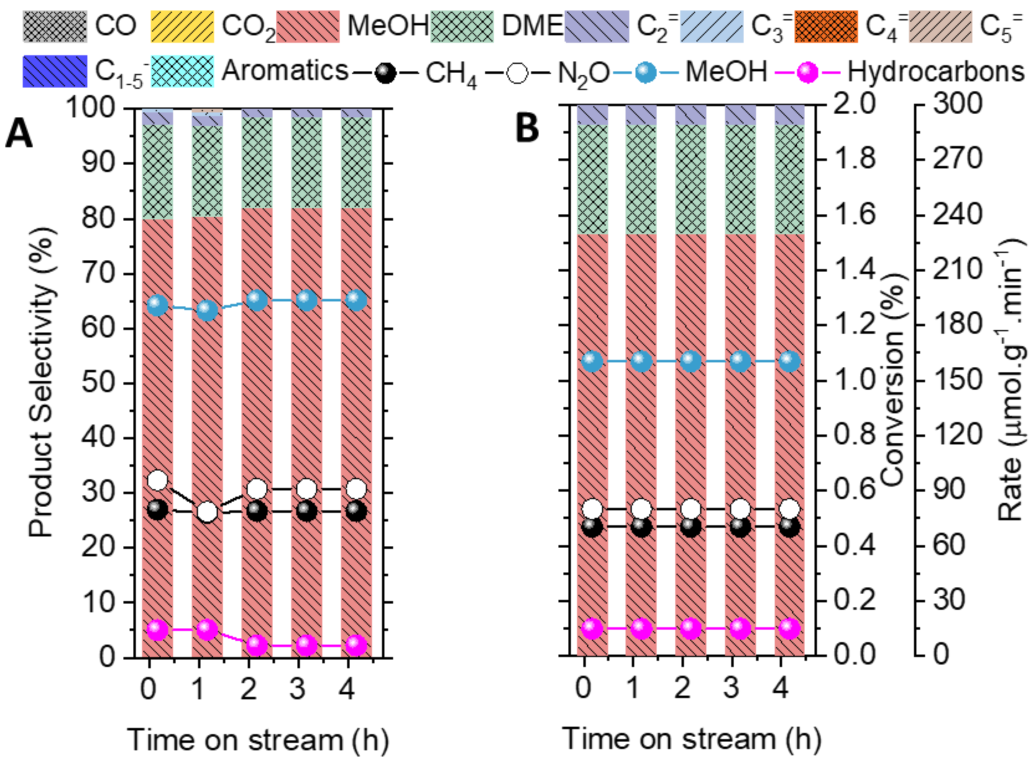

Figure S32. Stability test of (A) H-FER and (B) imp-5Al/FER. Reaction conditions: 10 mg catalyst, 350 ºC, $CH_4/N_2O/H_2O/Ar$ = 10/10/2/3 ml·min^{-1}, WHSV =15000 ml·g^{-1}·h^{-1}.

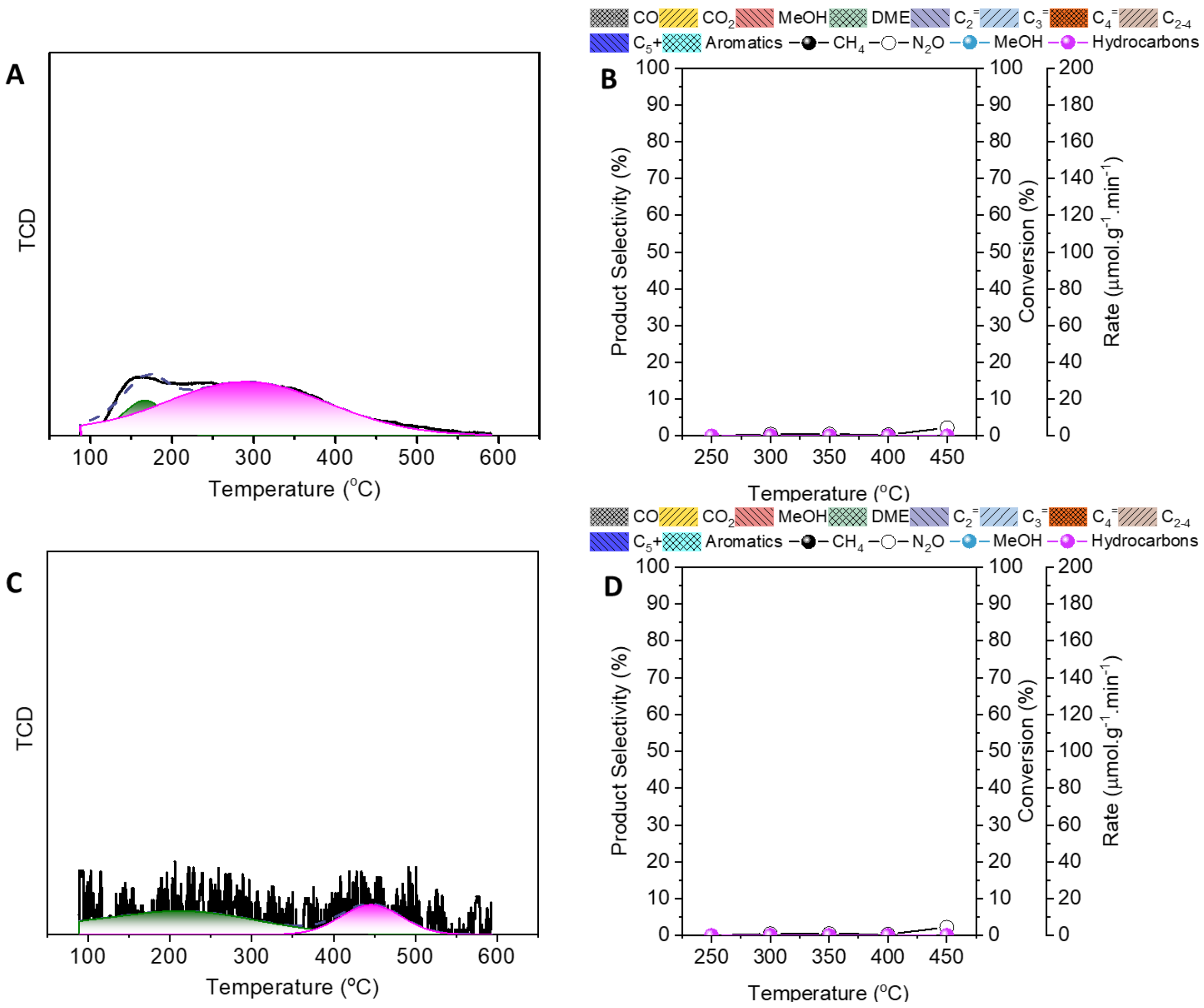

Figure S33. (A) NH_3-TPD curve of γ-Al_2O_3. (B) Catalytic performance of γ-Al_2O_3 at 250-450 °C after activation at 500 °C for 1 h, reaction conditions: 100 mg catalyst, $CH_4/N_2O/H_2O/Ar$ = 10/10/2/3 ml·min^{-1}, WHSV =15000 ml·g^{-1}·h^{-1}. (C) NH_3-TPD curve of α-Al_2O_3. (D) Catalytic performance of α-Al_2O_3 at 250-450 °C after activation at 500 °C for 1 h, reaction conditions: 100 mg catalyst, $CH_4/N_2O/H_2O/Ar$ = 10/10/2/3 ml·min^{-1}, WHSV =15000 ml·g^{-1}·h^{-1}.

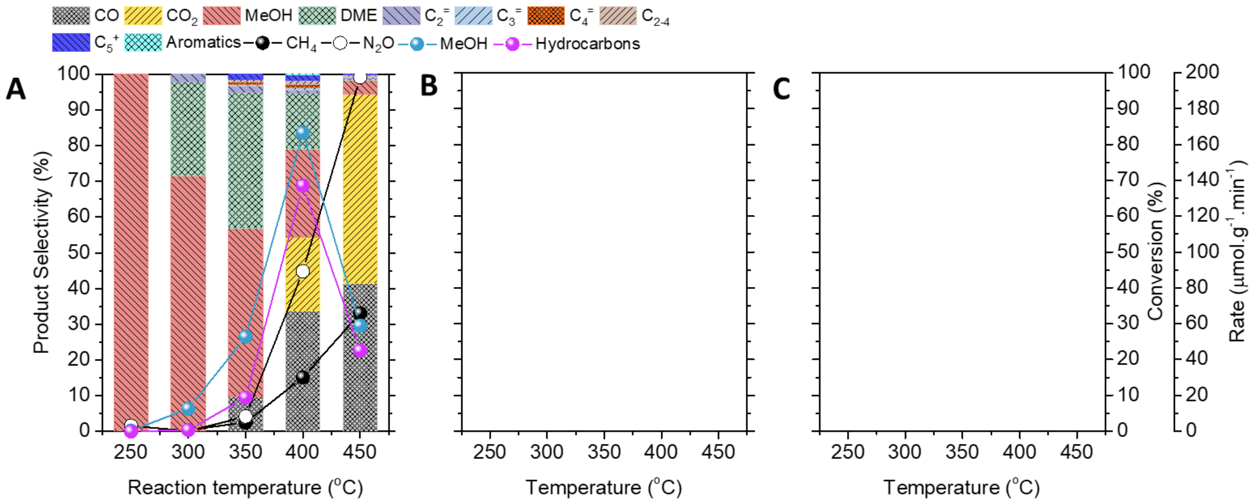

Figure S34. Catalytic performance of 100 mg H-FER at 250-450 ºC after activation at 500 ºC for 1 h using (A) N_2O ($CH_4/N_2O/H_2O/Ar$=10/10/2/3 ml·min^{-1}), (B) O_2 ($CH_4/O_2/H_2O/Ar$=10/10/2/3 ml min^{-1}), and (C) H_2O (CH_4/ H_2O/Ar=10/10/5 ml·min^{-1}) as the oxidant.

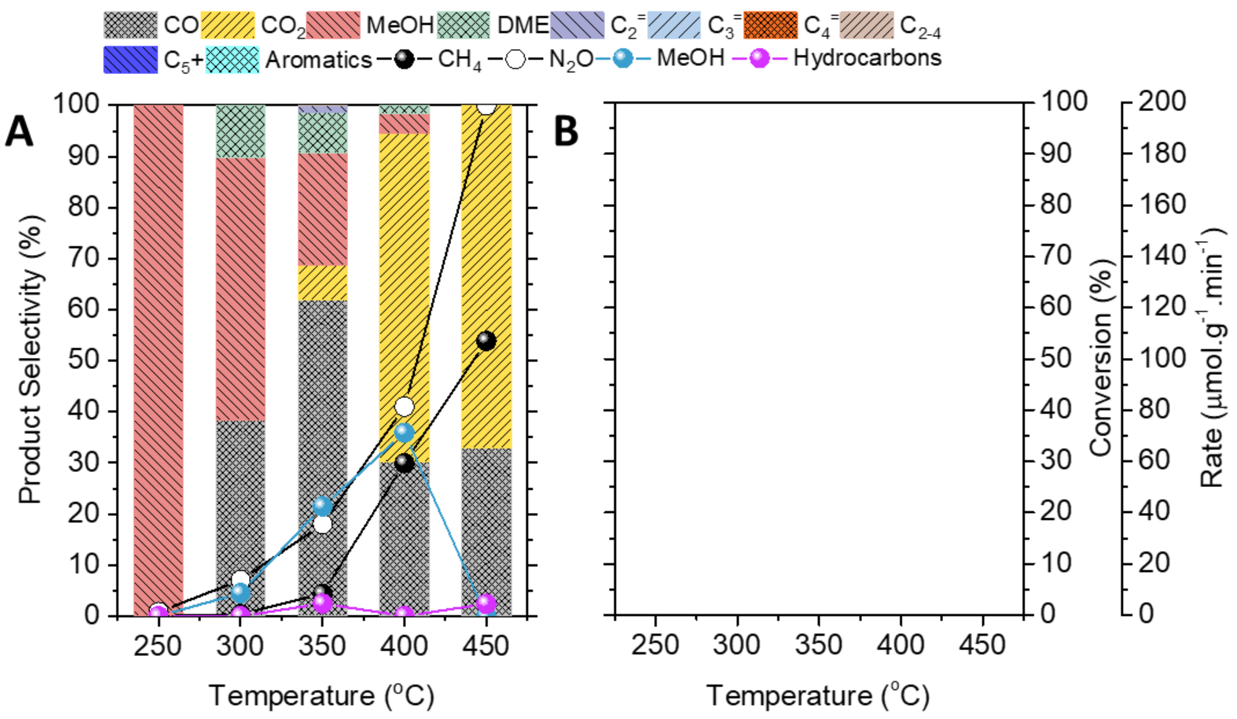

Figure S35. Catalytic performance of 100 mg 5Fe/FER at 250-450 °C after activation at 500 °C for 1 h using (A) N_2O ($CH_4/N_2O/H_2O$/Ar=10/10/2/3 ml·min^{-1}) and (B) O_2 ($CH_4/O_2/H_2O$/Ar=10/10/2/3 ml·min^{-1}) as the oxidant.

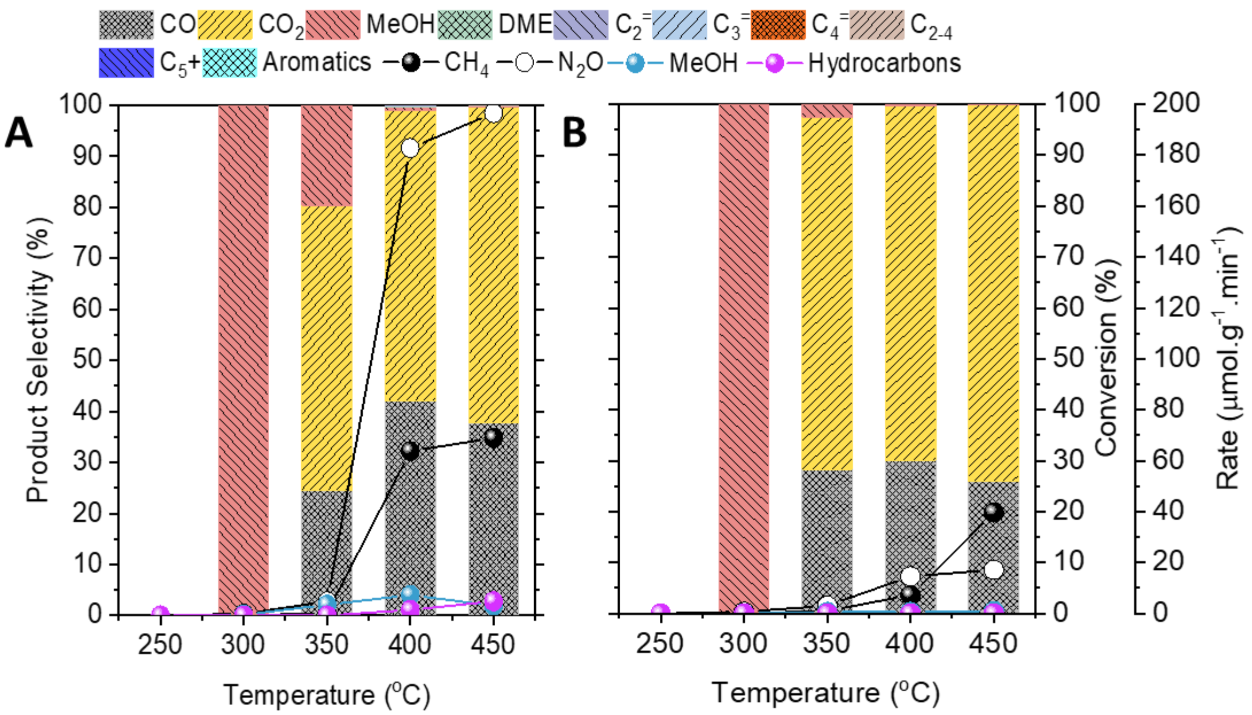

Figure S36. Catalytic performance of 100 mg 5Cu/FER at 250-450 °C after activation at 500 °C for 1 h using (A) N_2O ($CH_4/N_2O/H_2O/Ar=10/10/2/3$ ml·min^{-1}) and (B) O_2 ($CH_4/O_2/H_2O/Ar=10/10/2/3$ ml·min^{-1}) as the oxidant.

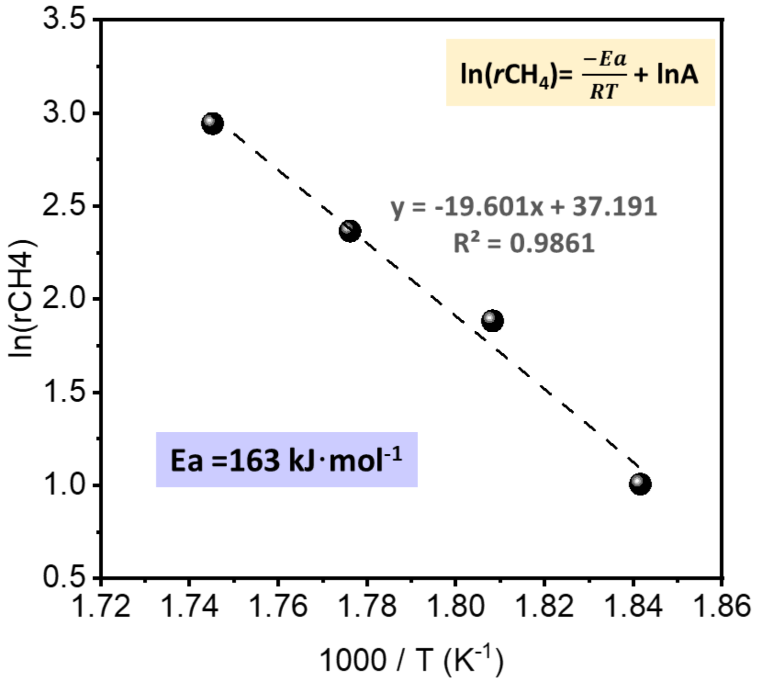

Figure S37. Apparent activation energy calculated at 270-300 ºC based on the Arrhenius formula. Reaction condition: 10 mg H-FER, 270-300 ºC , $CH_4/N_2O/H_2O/Ar$ = 10/10/2/3 ml·min^{-1}.

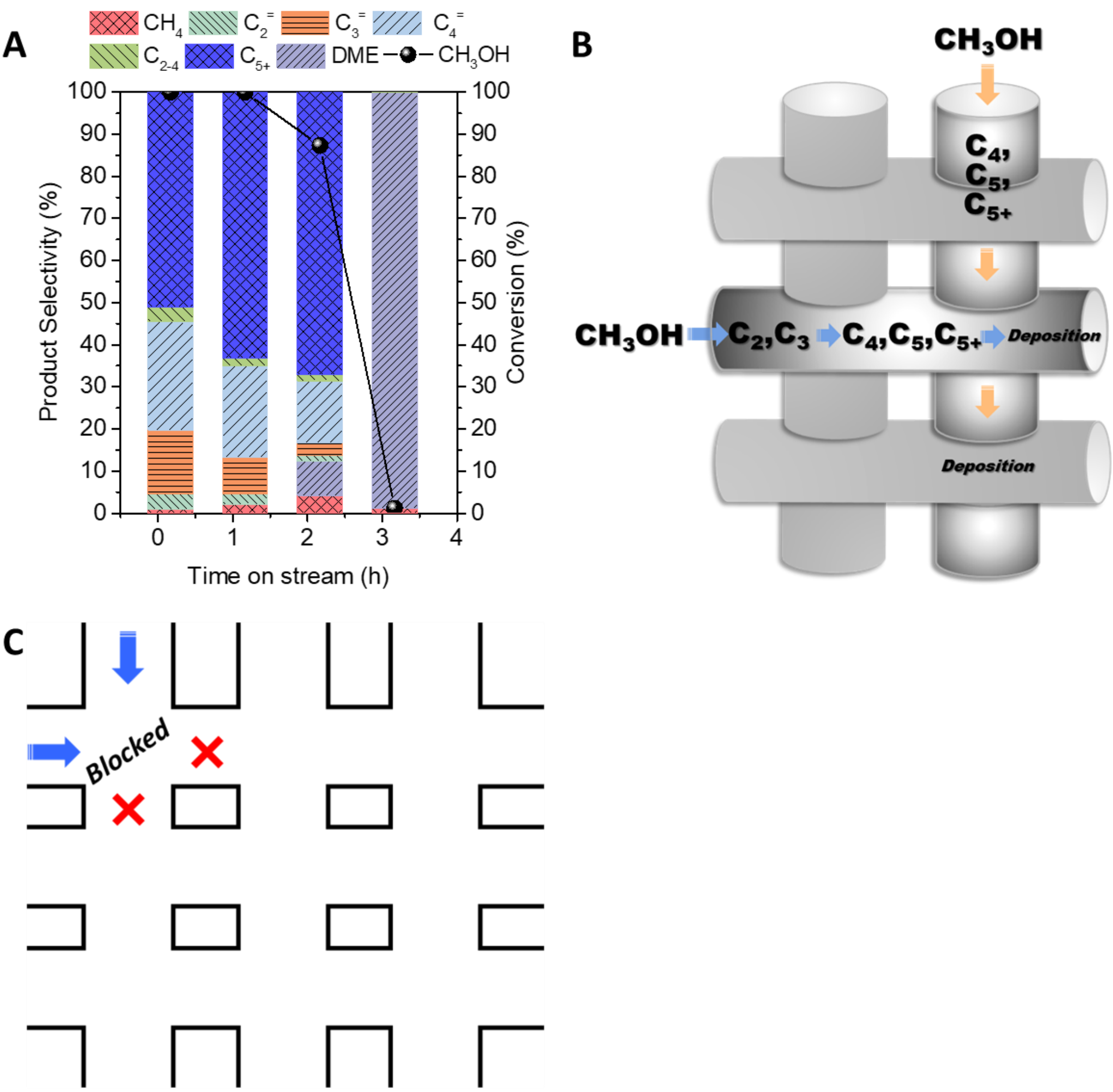

Figure S138. (A) Time courses of the MTH reaction at 350 °C on H-FER zeolite catalyst. Reaction condition: 100 mg catalyst, 5 vol% methanol in Ar gas, W/F_{MeOH} = 68 g·h·mol^{-1}. (B) The possbile formation process of different products in 8 and 10 ring channels. (C) Schematic diagram of a blockage at an intersection and the impassability of the connected channels.

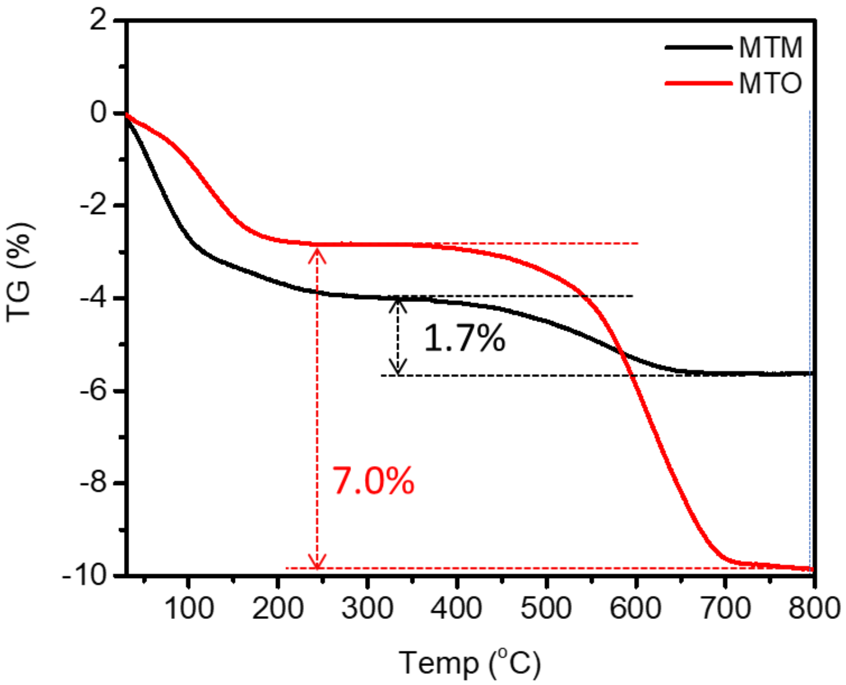

Figure S39. Weight loss based on TG curves of H-FER after DMTM at 350 °C for 12 h (Figure S6), 100 mg catalyst, $CH_4/N_2O/H_2O/Ar$ = 10/10/2/3 ml·min^{-1}, WHSV =15000 ml·g^{-1}·h^{-1}. And after MTH reaction at 350 °C for 4 h (Figure S38A), 100 mg catalyst, 5 vol% methanol in Ar gas, W/F_{MeOH} = 68 g·h·mol^{-1}.

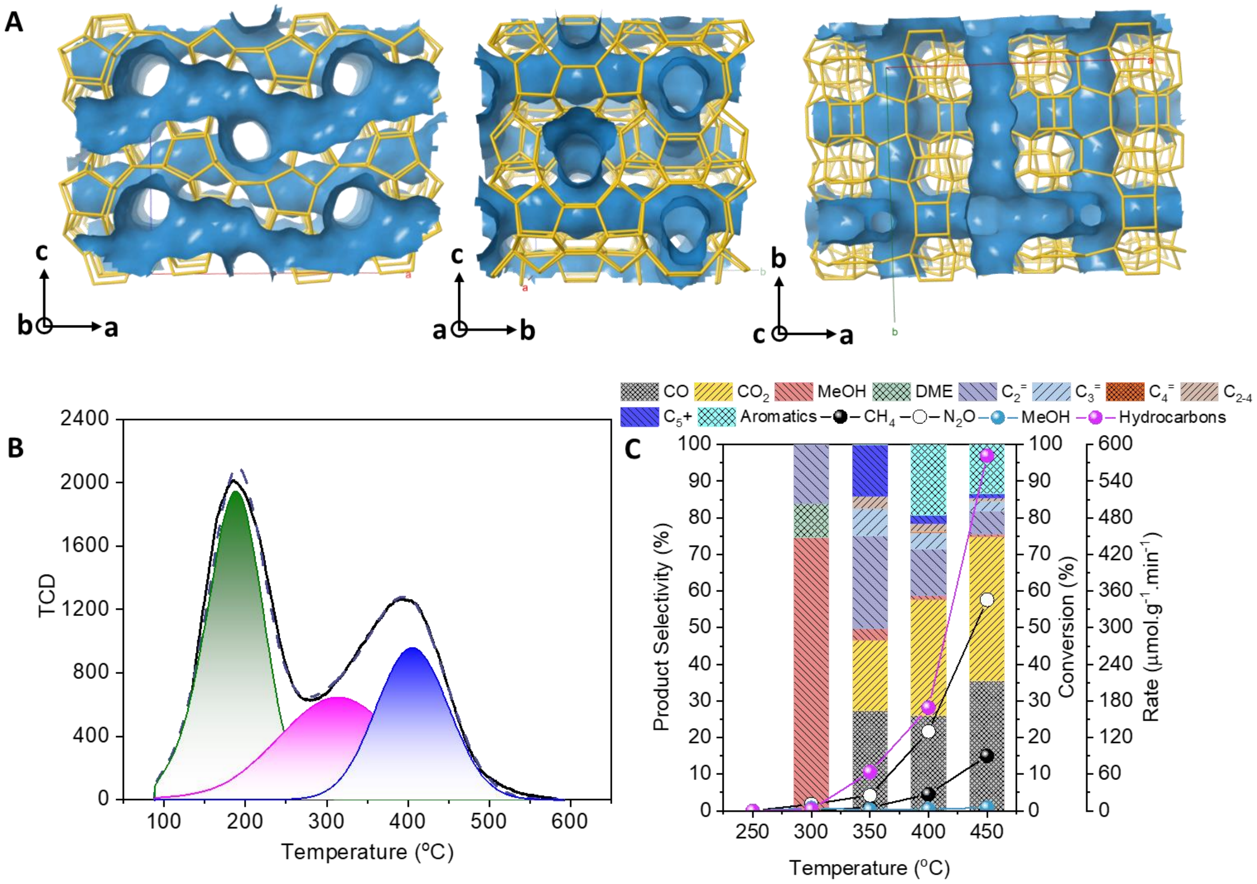

Figure S40. (A) MFI-type framework structure via b, a, c direction, respectively. (B) Deconvolution of NH_3-TPD curve for H-ZSM-5 (JRC-Z5-30NH4). (C) Catalytic performance of ZSM-5 continuous reaction at 250-450 °C after activation at 500 °C for 1 h, reaction conditions: 100 mg catalyst, $CH_4/N_2O/H_2O/Ar$=10/10/2/3 ml·min^{-1}, WHSV=15000 ml·g^{-1}·h^{-1}.

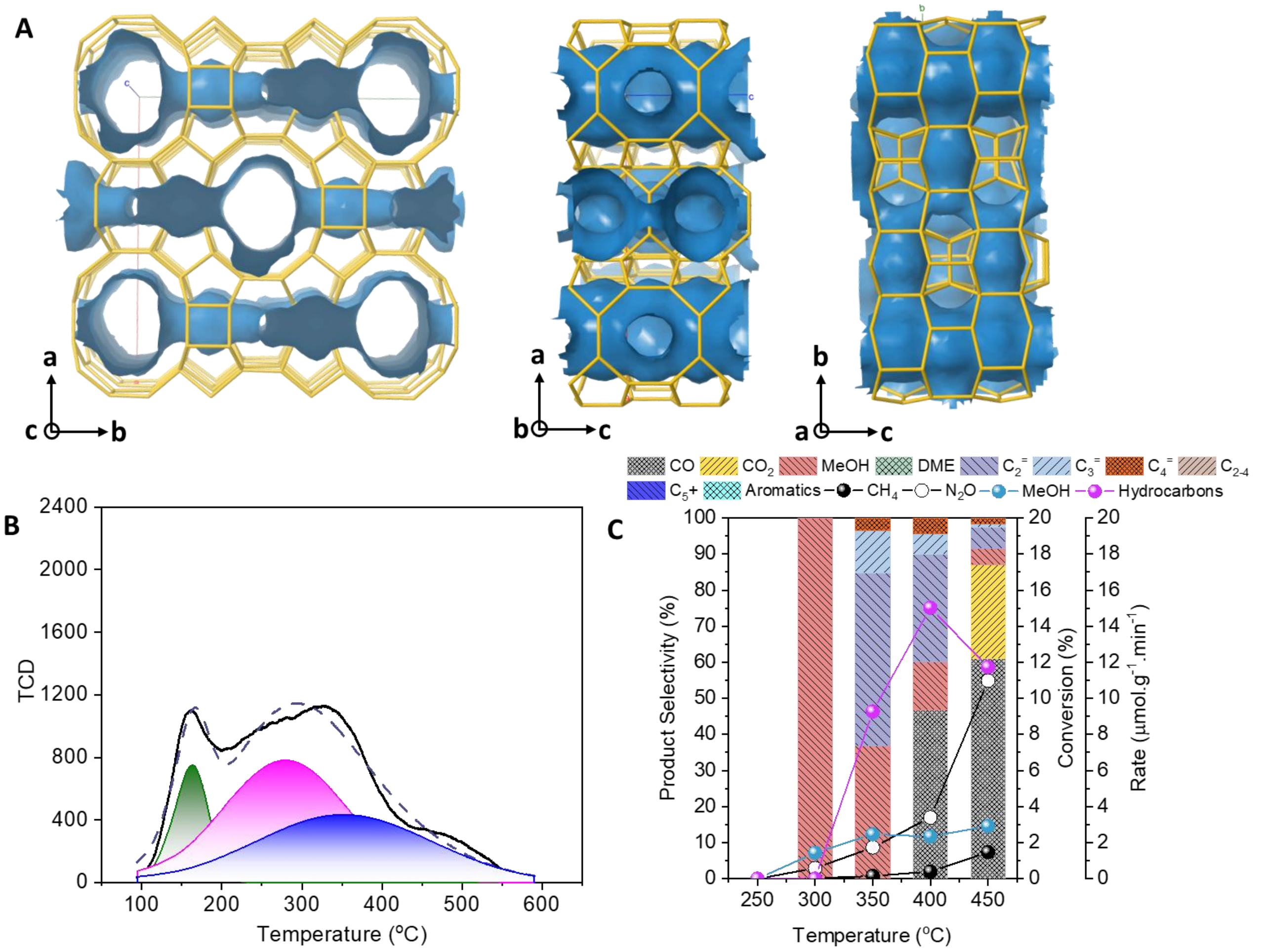

Figure S41. (A) MOR-type framework structure via c, b, a direction, respectively. (B) Deconvolution of NH_3-TPD curve for H-MOR (JRC-Z-HM20). (C) Catalytic performance of H-MOR zeolite continuous reaction at 250-450 °C after activation at 500 °C for 1 h, reaction conditions: 100 mg catalyst, $CH_4/N_2O/H_2O/Ar$=10/10/2/3 ml·min^{-1}, WHSV=15000 ml·g^{-1}·h^{-1}.

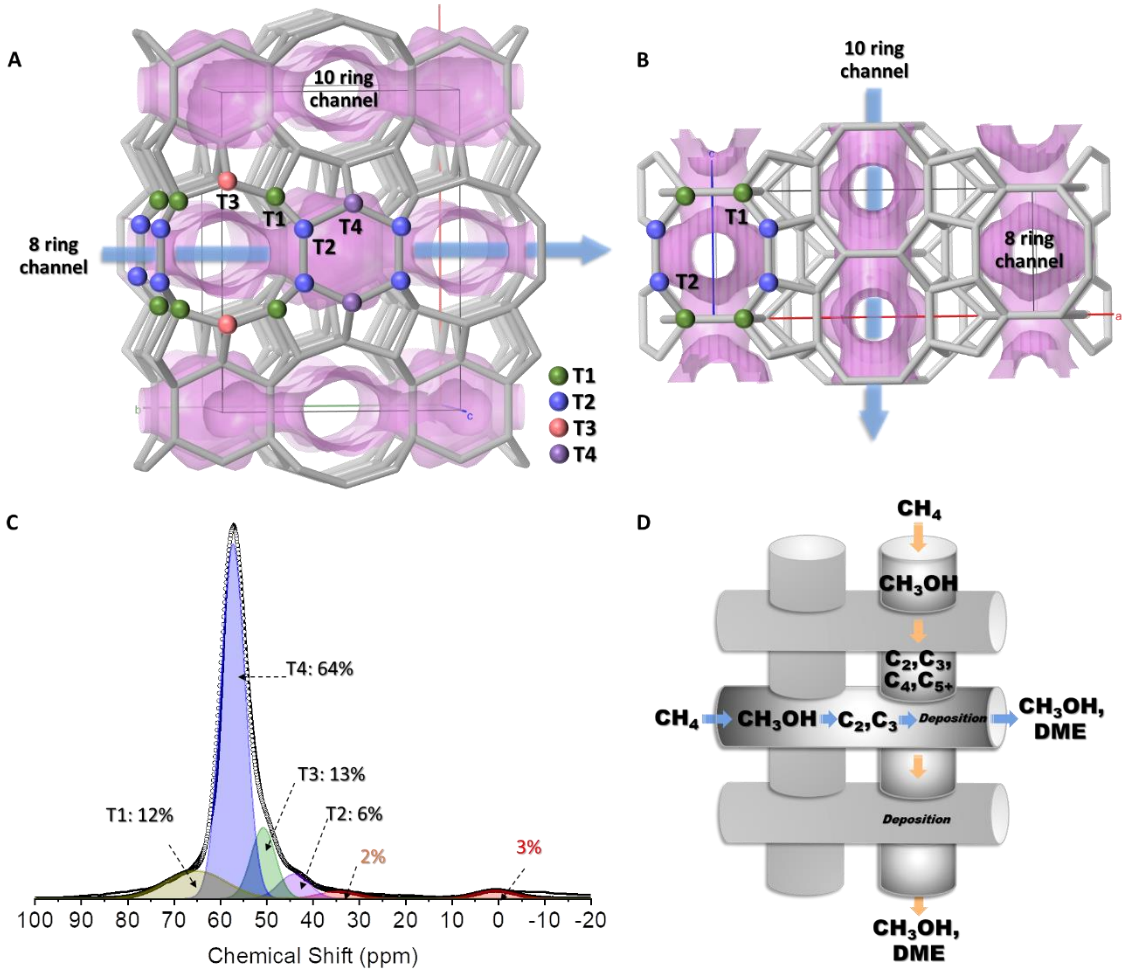

Figure S42. FER-type framework structure. The positions of the four crystallographically independent T sites, and the two perpendicular intersecting channels accessible via (A) 001 and (B) 010 faces. (C) Deconvolution of ^{27}Al MAS NMR spectra and the proportion at corresponding T site for (C) H-FER(Zeolyst) and (D) the possbile formation process of different products in 8 and 10 ring channels for the FER zeolite (CP914C, Zeolyst).

Table S1. Comparison the reaction performance of oxidation of methane to methanol reported in the literature.

**No.**	**Type**	**Catalyst**	**MeOH formation rate**	**MeOH selectivity**	**Reaction conditions**	**Reference**
1.	Continuous	H-FER-550	159 $\mu mol \cdot g^{-1} \cdot min^{-1}$	65 % (MeOH+DME > 97%)	1. 25 mg<br>2. 350 ºC<br>3. $CH_4/N_2O/H_2O$	This work
2.		H-FER-550	47 $\mu mol \cdot g^{-1} \cdot min^{-1}$	51 % (MeOH+DME > 88%)	1. 100 mg<br>2. 350 ºC<br>3. $CH_4/N_2O/H_2O$	This work
3.		H-FER-850	80 $\mu mol \cdot g^{-1} \cdot min^{-1}$	45 % (MeOH+DME > 98%)	1. 100 mg<br>2. 350 ºC<br>3. $CH_4/N_2O/H_2O$	This work
4.		H-FER-850	305 $\mu mol \cdot g^{-1} \cdot min^{-1}$	88 % (MeOH+DME > 99%)	1. 25 mg<br>2. 350 ºC<br>3. $CH_4/N_2O/H_2O$	This work
5.		H-ZSM-5	3.5 $\mu mol \cdot g^{-1} \cdot min^{-1}$	83 % (MeOH+DME > 90%)	1. 100 mg<br>2. 300 ºC<br>1. $CH_4/N_2O/H_2O$	This work
6.		H-MOR	2.5 $\mu mol \cdot g^{-1} \cdot min^{-1}$	100 %	3. 100 mg<br>4. 300 ºC<br>5. $CH_4/N_2O/H_2O$	This work
7.		5Cu/FER	10 $\mu mol \cdot g^{-1} \cdot min^{-1}$	15 %	1. 100 mg<br>2. 350 ºC<br>3. $CH_4/N_2O/H_2O$	This work

8.		5Fe/FER	3 μmol·g^{-1}·min^{-1}	0.2 %	1. 100 mg<br>2. 350 ºC<br>3. $CH_4/N_2O/H_2O$	This work
9.	Continuous	Cu/AEI	27 μmol·g^{-1}·min^{-1}	50 %	1. 100 mg catalyst<br>2. 350 ºC<br>3. $CH_4/N_2O/H_2O$	Appl. Catal. B, 2023, 325: 122395.
10.		Cu/AEI	41 μmol·g^{-1}·min^{-1}	45 %	1. 100 mg catalyst<br>2. 350 ºC<br>3. $CH_4/N_2O/H_2O$	ACS Catal. 2023, 13, 11057−11068.
11.		Fe-AEI	45 μmol·g^{-1}·min^{-1}	8 %	1. 100 mg catalyst<br>2. 350 ºC<br>3. $CH_4/N_2O/H_2O$	ACS Catal. 2023, 13, 16168−16178
12.		Cu/BEA	4 μmol·g^{-1}·min^{-1}	72 %	1. 500 mg catalyst<br>2. 320 ºC<br>3. $CH_4/N_2O/H_2O$	Angew. Chem., 2021, 133(30): 16770-16776.
13.		Cu/AEI	8 μmol·g^{-1}·min^{-1}	34 %	1. 300 mg catalyst<br>2. 300 ºC<br>3. $CH_4/N_2O/H_2O$	Chem.Comm.2021, 57(11): 1364-1367.
14.		Fe/FER	-	-	1. $CH_4/N_2O/H_2O$	1. ACS Catal. 2020, 10, 1406−1416<br>2. J. Catal. 2023, 417,140–152<br>3. J. Catal.2021, 400,10–19

15.		Cu/CHA	-	13 %	1. 300 mg catalyst<br>2. 300 ºC<br>2. $CH_4/N_2O/H_2O$	Chem.Comm., 2016, 52(91): 13401-13404.
16.		Fe-MFI	0.9 $\mu mol \cdot g^{-1} \cdot min^{-1}$	-	1. 360 mg catalyst<br>2. 275 ºC<br>3. $CH_4/N_2O/H_2O$	J. Catal. 2014, 318,14–21
17.		Cu/AEI	0.1 $\mu mol \cdot g^{-1} \cdot min^{-1}$	-	1. 400 mg catalyst<br>2. 225 ºC<br>3. $CH_4/O_2/H_2O$	J. Catal., 2023, 421,300-308
18.		Cu/CHA	3.3 $\mu mol \cdot g^{-1} \cdot min^{-1}$	91 %	1. 100 mg catalyst<br>2. 300 ºC<br>3. $CH_4/O_2/H_2O$	Chem, 2021,7,1557-1568
19.		Cu/CHA	0.3 $\mu mol \cdot g^{-1} \cdot min^{-1}$	53 %	1. 270 ºC<br>2. $CH_4/O_2/H_2O$	JACS 2019,141, 11641
20.		Cu-Na-ZSM-5	0.03 $\mu mol \cdot g^{-1} \cdot min^{-1}$	71 %	1. 210 ºC<br>2. $CH_4/O_2/H_2O$	ACS Cent. Sci. 2016, 2, 6, 424–429
21.	Stepwise	Cu-MOF-88	70 $\mu mol \cdot g^{-1}$	-	i) 150 ºC in N_2O (activation)<br>ii) 150 ºC in CH_4 (reaction)<br>iii) 150 ºC in H_2O (extraction)	JACS 2018,140, 18208

22.		Cu/MOR	97 μmol ·g^{-1}	-	i) 600 ºC in N_2O (activation)<br>ii) 150 ºC in CH_4 (reaction)<br>iii) 135 ºC in H_2O (extraction)	Chem. Common. 2017,53, 4116
23.		Cu/Al_2O_3	8 μmol ·g^{-1}	83 %	i) 427 ºC in air (activation)<br>ii) 200 ºC in CH_4 (reaction)<br>iii) 25 ºC in H_2O (extraction)	Angew. Chem.2019, 131, 9946
24.		Cu-MOR	155 μmol ·g^{-1}	90 %	i) 450 ºC in O_2 (activation)<br>ii) 200 ºC in CH_4 (reaction)<br>iii) 200 ºC in H_2O (extraction)	ACS Catal.2019, 9, 5308
25.		Cu/SiO_2	12 μmol ·g^{-1}	-	i) 800 ºC in O_2 (activation)<br>ii) 200 ºC in CH_4 (reaction)<br>iii) 200 ºC in H_2O (extraction)	ACS Catal.2018, 8, 5721
26.		Cu-NU-1000	18 μmol ·g^{-1}	45 %	i) 200 ºC in O_2 (activation)<br>ii) 150 ºC in CH_4	JACS 2017,139, 10294

					(reaction)<br>iii) 135 ºC in H_2O (extraction)	
27.		Cu-CHA	28 μmol·g^{-1}	95 %	i) 723 K in O_2 (activation)<br>ii) 323 K in CH_4 (reaction)<br>iii) 473 K in H_2O (extraction)	ACS Catal.2017, 7, 4291
28.		Cu-CHA	125 μmol·g^{-1}	90 %	i) 500 ºC in O_2 (activation)<br>ii) 200 ºC in CH_4 (reaction)<br>iii) 200 ºC in H_2O (extraction)	JACS 2017,139, 14961
29.		Cu-MOR	204 mmol·mol_{Me}^{-1}	97 %	i) 400 ºC in He (activation)<br>ii) 200 ºC in CH_4 (7bar)(reaction)<br>iii) 200 ºC in H_2O (7bar) (extraction)	Science 2017,356, 523
30.		Cu-MOR	103 μmol·g^{-1}	-	i)450 ºC in He (activation)<br>ii)200 ºC in CH_4(36 bar) (reaction)<br>iii) 200 ºC in H_2O (extraction)	Angew. Chem.2016, 128, 5557

31.		Cu-MOR	160 μmol ·g^{-1}	80 %	i) 550 ºC in He (activation)<br>ii) 200 ºC in CH_4 (reaction)<br>iii) 135 ºC in H_2O (extraction)	Nat. Commun. 2015, 6, 7546
32.		pMMO	1380 μmol ·g^{-1}	-	i) Duroquinol as electrondonor;<br>ii) 40 ºC in CH_4(reaction)	Nature 2010, 464, 115
33.		Cu-MFI	9 μmol ·g^{-1}	98 %	i) 450 ºC in O_2 (activation)<br>ii) 175 ºC in CH_4 (reaction)<br>iii) RT in 1:1 H_2O/CH_3CN (extraction)	JACS,2005,127,1394
34.		Cu/FAU	360 μmol ·g^{-1}	-	i)400 ºC in O_2 (activation)<br>ii)360 ºC in CH_4(15 bar) (reaction)<br>iii) 25 ºC in H_2O (extraction)	ACS Catal. 2019, 9, 7, 6293.

Table S2. Chemical composition and acidity of zeolites.

Sample	Chemical Compositions [a]		Acidity by NH_3-TPD ($mmol\cdot g^{-1}$) [c]			
	Si/Al	M/Al(wt.%) [b]	Weak	Medium	Strong	Total
H-FER	29.0	-	0.39	0.24	0.38	1.01
Na/FER	29.0	0.88	0.17	0.44	0.17	0.79
K/FER	28.6	0.61	0.27	0.06	-	0.33
0.05Fe/FER	28.1	0.006 (0.02)	0.35	0.21	0.27	0.83
0.5Fe/FER	28.0	0.062 (0.2)	0.27	0.19	0.22	0.69
1Fe/FER	28.0	0.15 (0.47)	0.31	0.30	0.21	0.82
5Fe/FER	28.3	0.82 (2.5)	0.22	0.16	0.35	0.73
0.1Cu/FER	28.0	0.002 (0.01)	0.32	0.23	0.26	0.81
1Cu/FER	28.0	0.02 (0.09)	0.27	0.26	0.22	0.75
5Cu/FER	28.2	0.11 (0.36)	0.23	0.14	0.37	0.74
50Cu/FER	28.0	0.74 (2.6)	0.22	0.16	0.37	0.75
H-FER-750	29.0	-	0.22	0.13	0.28	0.62
H-FER-850	29.0	-	0.10	0.09	0.28	0.48
H-FER-950	29.0	-	0.08	0.10	0.22	0.40
DeAl-FER-0.5	73.0	-	0.18	0.14	0.15	0.46
DeAl-FER-1.0	558.9	-	0.02	0.02	-	0.04
Imp-5Al/FER	15.2	-	0.27	0.49	0.66	1.42
H-FER(Pyrr)	8.8	-	0.76	0.41	0.46	1.63
H-ZSM-5	16.5	-	0.64	0.45	0.41	1.50
H-MOR	10.0	-	0.14	0.59	0.46	1.19

[a] by ICP-AES.
[b] M means metal, including Na, K, Fe, Cu.
[c] by NH_3-TPD; fitting curves of the weak, medium, strong acid sites were calculated at approximately 160, 300 and 475 °C, respectively.

Table S3. Al distribution of fresh FER zeolites.

Sample	Total Al distribution (%)				Framework Al distribution (%)			
	Al_{IV-1}	Al_{IV-2}	Al_{V}	Al_{VI}	Al_{T1}	Al_{T2}	Al_{T3}	Al_{T4}
	63~50 ppm	> 63, 50~40 ppm	40-20 ppm	20~(-20) ppm	60 ppm	50 ppm	54 ppm	57 ppm
NH_4-FER	100	0	0	0	6	14	41	39
H-FER-550 (H-FER) (H-FER(Zeolyst))	80	16	2	2	13	6	14	67
H-FER-750	62	20	7	11	-	-	-	-
H-FER-850	37	16	26	21	-	-	-	-
H-FER-950	34	15	20	31	-	-	-	-
DeAl-FER-0.5	81	4	4	11	-	-	-	-
DeAl-FER-1.0	77	15	6	2	-	-	-	-
Imp-5Al/FER	70	10	9	11	-	-	-	-
H-FER(Pyrr)	91		6	3	11	10	36	43

Table S4. Textual properties of FER zeolites.

Sample	Textual Properties			
	$S_{BET}(m^2 \cdot g^{-1})$ [a]	$V_{Total}(cm^3 \cdot g^{-1})$ [a]	$S_{EXT}(m^2 \cdot g^{-1})$ [b]	$V_{Mic}(cm^3 \cdot g^{-1})$ [b]
H-FER (H-FER-550) (H-FER(Zeolyst))	329	0.30	33	0.11
H-FER-750	367	0.29	23	0.13
H-FER-850	374	0.32	27	0.14
H-FER-950	397	0.34	20	0.13
DeAl-FER-0.5	160	0.35	30	0.05
DeAl-FER-1.0	34	0.17	21	0.01
Imp-5Al/FER	324	0.26	30	0.14
H-FER(Pyrr)	378	0.30	37	0.15

[a] by the Brunauer–Emmett–Teller (BET) equation on the N_2 adsorption isotherms.

[b] by the t-plot method based on the adsorption isotherms.

Reference

[1] Dai, W.; Ruaux, V.; Deng, X.; Tai, W.; Wu, G.; Guan, N.; Li, L.; Valtchev, V., Synthesis and catalytic application of nanorod-like FER-type zeolites. *Journal of Materials Chemistry A* **2021,** *9* (44), 24922-24931.
